# High-sensitivity spin-exchange relaxation-free (SERF) magnetometry combined with magnetic-responsive iron oxide nanoparticles for real-time monitoring of malignant tumor therapy

**DOI:** 10.7150/thno.117893

**Published:** 2026-01-01

**Authors:** Wenbo Wang, Gaorui Zhang, Baosheng Wang, Dawei Zhou, Ning Ding, Ziyuan Huang, Shiqiang Zheng, Qichao Cheng, Mingchuan Yu, Min Xiang, Yuguo Chen, Jiazhi Duan, Dexin Yu

**Affiliations:** 1 Department of Radiology, Qilu Hospital of Shandong University, Jinan, Shandong, 250012, China.; 2 Department of Radiology, Weifang People's Hospital, Shandong Second Medical University, Weifang, China.; 3 National Institute of Extremely-Weak Magnetic Field Infrastructure, Hangzhou 310028, China.; 4 School of Instrumentation and Optoelectronic Engineering, Beihang University, Beijing 100083, China.; 5 Institute for Advanced Interdisciplinary Research, University of Jinan, Jinan 250022, China.; 6 Department of Rehabilitation Medicine, the Second Affiliated Hospital of Nanchang University, Nanchang 330006, P. R. China.; 7 Interdisciplinary Center, Shandong University, Jinan 250100, China.; 8 Department of Emergency Medicine, Qilu Hospital of Shandong University, Jinan 250012, China.

**Keywords:** magnetic relaxometry, Fe₃O₄ nanoparticles, spin-exchange relaxation-free magnetometer, ultraweak magnetic field, malignant tumor

## Abstract

**Rationale**: Early and accurate evaluation of chemotherapy efficacy remains essential, yet conventional imaging approaches rely on delayed morphological changes. Functional alterations such as apoptosis and reduced metabolic activity occur earlier but are difficult to detect noninvasively. Magnetic signal detection offers a promising alternative but is limited by signal instability and biological noise.

**Methods**: We developed a magnetic signal-based monitoring platform by combining magnetically responsive ferromagnetic-superparamagnetic iron oxide nanoparticle (F-SPION) with a spin-exchange relaxation-free magnetometer, with signal amplification achieved through rubidium magnetization. *In vitro*, we assessed the linear correlation between magnetic signal intensity and tumor cell number, and further evaluated doxorubicin (DOX)-induced signal changes under constant cell conditions. Prussian blue staining was used to confirm changes in F-SPION uptake. *In vivo*, F-SPION was intravenously injected into tumor-bearing mice, and magnetic signals from tumor and normal tissues were measured at multiple time points after magnetization. The mice were randomly assigned to control or doxorubicin-treated groups, and tumor signals were monitored on Days 1, 7, 14, and 21. Biocompatibility was assessed through cytotoxicity, hemolysis, histology, and blood analysis.

**Results**: *In vitro*, magnetic signal intensity strongly linearly correlated with tumor cell number (R² = 0.974). Doxorubicin treatment resulted in signal reduction despite the identical cell numbers (control: 267.88 ± 5.97 pT; 24 h: 206.02 ± 2.23 pT; 48 h: 122.74 ± 2.11 pT), with Prussian blue staining confirming reduced F-SPION uptake. *In vivo*, the signal peaked at 0.5 h post-injection (1528.54 ± 23.34 pT). The tumor signals were consistently greater than the signals of normal tissues at 5 min (802.7 ± 60.8 vs. 149.3 ± 16.2 pT) and 60 min (163.6 ± 3.2 vs. 42.8 ± 1.5 pT). On Day 1, the signal of the treatment group was 425.3 ± 24.4 pT and remained stable until Day 7 (425.4 ± 14.4 pT), whereas that of the control group increased from 481.4 ± 3.8 to 830.7 ± 5.9 pT.

**Conclusions**: This magnetic signal-based platform enables noninvasive, real-time, and functional monitoring of tumor response, offering a sensitive and translational strategy for early-phase therapeutic evaluation.

## Introduction

Chemotherapy remains a cornerstone of cancer treatment, with 60%-80% of patients receiving it depending on tumor type and stage 1,2. Timely adjustment of therapeutic strategies is essential for improving clinical outcomes, as chemotherapy-induced changes in tumor cell viability and drug resistance cause treatment efficacy to vary over time 3-6. Consequently, conventional monitoring based on pre- and post-treatment assessments fails to support timely clinical intervention 7. Therefore, real-time monitoring of therapeutic response is critical, yet existing tools face significant limitations. Magnetic resonance imaging (MRI) and computed tomography (CT), although convenient and noninvasive 8-11, lack the sensitivity to detect early cellular functional changes 12,13, pathology provides functional insights but is invasive and impractical for repeated sampling 14-17, and positron emission tomography (PET) imaging, despite providing functional insights, is limited by radiation exposure and high cost 18-21.

Spin-exchange relaxation-free (SERF) magnetometry is an ultrasensitive, noninvasive technique capable of detecting extremely weak magnetic fields generated by bioelectric currents associated with cellular metabolic activities 22-25. Owing to their metabolic origin, these magnetic signals reflect the real-time functional states of cells 26-29, making SERF an attractive tool for assessing chemotherapy efficacy by monitoring apoptosis-induced magnetic changes in tumor cells. SERF offers advantages such as real-time detection, radiation-free operation, and high sensitivity to functional cellular changes 30,31. The weak and rapidly fluctuating magnetic signals in tumor tissues, coupled with their complex and heterogeneous composition 32-34, limit the sensitivity and accuracy of SERF in therapeutic monitoring.

Magnetic materials such as iron offer a promising strategy for enhancing the sensitivity of SERF-based tumor monitoring by amplifying weak magnetic signals from biological tissues 35,36. Among them, superparamagnetic iron oxide nanoparticles (SPIONs) are widely used because they exhibit no magnetism in the absence of an external field, which helps minimize background interference. Their magnetic response, although initially effective, diminishes rapidly after magnetization, limiting their ability to support sustained detection in ultraweak magnetic environments 37,38. By contrast, ferromagnetic materials retain magnetization over extended periods, enabling continuous signal acquisition, but their strong remanence introduces significant background noise, reducing the signal-to-noise ratio and detection specificity 39. Therefore, selecting materials that offer both magnetic stability and low background noise is essential for improving the sensitivity of SERF-based therapeutic monitoring.

In this study, we employed F-SPION, a magnetic material that combines both ferromagnetic and superparamagnetic properties, and can sustain intracellular magnetization after cellular uptake and subsequent magnetic activation, to enhance the sensitivity of SERF-based therapeutic monitoring under zero-field conditions. F-SPION was synthesized via a controlled coprecipitation method using FeCl₂ and FeCl₃ as precursors under an argon atmosphere. The improved sensitivity of SERF detection is attributed to the magnetic duality of F-SPION: on the one hand, the weak ferromagnetic property of F-SPION ensures prolonged magnetic retention after activation; on the other hand, its superparamagnetic nature minimizes background noise before magnetization, thereby enhancing temporal stability and signal specificity. The correlation between magnetic signal variation and therapeutic response was validated at both the cellular and animal levels, demonstrating that changes in magnetic signals reliably reflect tumor cell number and nanoparticle uptake capacity during treatment. In summary, F-SPION, with dual magnetic behavior, combined with a high-sensitivity SERF magnetometer, enables highly sensitive monitoring of tumor therapeutic responses under zero-field conditions by capturing dynamic changes in magnetic signals from tumor cells. This magnetic-responsiveness-based strategy not only expands the application scope of SERF technology in tumor magnetic signal monitoring, but also provides a novel approach for therapeutic evaluation that is noninvasive, real-time, and functionally informative.

## Methods and Materials

### Experimental reagents

Ferrous chloride (catalog number: I837006), ferric chloride (catalog number: I811935), ammonia (catalog number: A801006), doxorubicin (DOX) hydrochloride (catalog number: D807083), and ferumoxytol (catalog number: 722492-56-0) were purchased from Shanghai Macklin Biochemical Co., Ltd., China. LPS (lipopolysaccharide, catalog number: L8880) and a Prussian blue staining kit (catalog number: G1422) were purchased from Solarbio, China. A Cell Counting Kit-8 (CCK-8) (catalog number: HY-K0301) was purchased from MCE, USA. Anti-CD31/PECAM1 antibody (catalog number: A01513-2) was purchased from Boster, China. A terminal deoxynucleotidyl transferase dUTP nick-end labeling (TUNEL) staining kit was purchased from UElandy, Suzhou, China (catalog number: T6013L).

### Experimental equipment

Optically pumped magnetometers (QZFM Gen2, QuSpin Inc., USA; field sensitivity: <15 fT/√Hz in the 3-100 Hz band, typical: 7-10 fT/√Hz; dynamic range: ±5 nT). Ultraweak magnetic field measurement chamber (permalloy, 5-layer shielding; noise level: <15 fT/√Hz in the uniform region). Transmission electron microscope (TEM) (JEM-1200EX, JEOL, Japan). MRI instrument (HDX TWINSP, GE, USA). X-ray diffraction (XRD) system (D8 Advance, Bruker, Germany). X-ray photoelectron spectrometer (XPS) (ESCALAB 250, Thermo Fisher Scientific, USA). Vibrating sample magnetometer (VSM) (MicroMag™ 2900 AGM, Lake Shore Cryotronics, USA). Fourier transform infrared spectrometer (FTIR) (Nexus 670, Thermo Fisher Scientific, USA). Inductively coupled plasma mass spectrometer (ICP-MS) (Agilent 7900, Agilent Technologies, USA). Dynamic light scattering (DLS) system (Zetasizer Nano ZS90, Malvern Instruments, UK). 16-channel mouse coil (MS160, Shenzhen Medcoil Healthcare Technology Co., Ltd., China).

### Nanomaterial preparation and characterization

FeCl_2_ (0.2 g) and FeCl_3_ (0.55 g) were added to 20 mL of deionized water. The solution was thoroughly mixed and heated to 80 °C under argon. Then, 5 mL of ammonia was added to the solution, and this mixture was maintained at 80 °C for 1 h. The obtained nanoparticles were magnetically collected and washed three times. The morphology and size distribution were characterized by transmission electron microscopy with statistical analysis, and the hydrodynamic diameter and polydispersity index (PDI) were determined by dynamic light scattering. The crystal structure was analyzed by X-ray diffraction, and the elemental composition and Fe oxidation states were analyzed by X-ray photoelectron spectroscopy. Magnetic properties were measured by vibrating sample magnetometry to obtain saturation magnetization (Ms), remanence (Mr), and coercivity (Hc). Surface functional groups were identified by Fourier transform infrared spectroscopy, with an emphasis on -OH stretching.

### Signal measurement and magnetization

For magnetic signal measurement, F-SPION and 4T1 cells were placed in T25 culture flasks; tumor-bearing mice were anesthetized with 1% pentobarbital (40 mg/kg) and fixed onto a polypropylene plastic board, and mouse organs were placed in tissue embedding boxes. All the samples were mounted on a motor-driven shaft rotating at 1.7 Hz inside a five-layer permalloy magnetic shielding chamber. An optically pumped magnetometer was positioned 1-2 mm beneath the test sample using a 3D-printed holder to record magnetic signals. During each rotation, the center of the magnetic signal detection area passed over the sensor once. Data were recorded at 200 Hz for 30 s. For magnetization, a rubidium magnet (10 × 10 × 5 cm, 0.15 T) was placed beneath the sample and gently rotated to ensure uniform magnetization of the target area, including F-SPION, 4T1 cells, tumor tissues, and major organs. For samples subjected to magnetization, unless otherwise specified, magnetic signals from F-SPION, 4T1 cells, tumor tissues, and major organs were measured 5 min after magnetization. A bandpass filter was applied to improve the signal-to-noise ratio. Fast Fourier transform (FFT) analysis revealed a consistent 1.7 Hz spectral peak, corresponding to rotational magnetic field modulation.

### Experimental cells and cell culture

Mouse triple-negative breast cancer 4T1 cells were purchased from the Type Culture Collection of the Chinese Academy of Sciences (China). Cells were cultured in 1640 medium supplemented with 10% fetal bovine serum and 500 IU/mL penicillin/streptomycin at 37 °C, 5% CO₂, and constant humidity.

### Cell viability assay

8000 4T1 cells per well were seeded in a 96-well plate and incubated at 37 °C with 5% CO₂ 40. After cell adhesion, the medium was replaced with fresh medium containing F-SPION at concentrations of 12.5, 25, 50, 100, and 200 µg/mL, and the cells were incubated for 24 h. Then, 100 µL of CCK-8 reagent was added to each well, the cells were incubated for an additional 2 h, and the absorbance was measured at 450 nm. The cell viability was calculated, and concentration‒effect curves were constructed.

### Animal model

BALB/c mice were purchased from Jinan Peng Yue Laboratory Animal Co., Ltd. (Jinan, China) and fed in accordance with laboratory standards. For tumor models, 4T1 cells (1 × 10⁶) were inoculated into the unilateral or bilateral inguinal region of female BALB/c mice 41. To establish excisional wound models, the mice were anesthetized with isoflurane (2%-4% in oxygen), and the fur was removed using an electric razor and depilatory cream. The skin was then excised with surgical scissors. After the procedure, the mice recovered in a warm environment 42.

### Magnetization-induced signal enhancement

To evaluate the responsiveness of F-SPION to external magnetic fields and identify factors influencing its signal intensity, magnetic signals were measured before and after 30 s of magnetization at various F-SPION concentrations (25, 50, 100, 200, and 400 µg/mL, dissolved in 3 mL of phosphate-buffered saline (PBS), with n = 5. Additionally, magnetic signals of F-SPION at a fixed concentration (100 µg/mL, 3 mL total volume) were recorded after different durations of magnetization (10, 20, 30, 60, and 120 s, n = 5).

### Evaluating the impact of cell-F-SPION interactions on magnetic signals

F-SPION-mediated magnetic signal enhancement is governed by magnetic relaxation mechanisms, which are modulated by cellular uptake and nanoparticle-cell interactions. To investigate the effect of nanoparticle-cell binding on signal enhancement, the following groups (n = 5) were established (each in a total sample volume of 3 mL): PBS: control group; 4T1 + 1640 medium: 4T1 cells (3.0 × 10⁶) were cultured in 1640 medium to assess the intrinsic magnetic response of cells in the absence of F-SPION; F-SPION+4T1: 4T1 cells (3.0 × 10⁶) were cultured in 1640 medium, and F-SPION (100 µg/mL, dissolved in 1640 medium) was introduced immediately before measurement, without allowing time for interaction; and F-SPION+4T1 co-culture: 4T1 cells (3.0 × 10⁶) were co-incubated with F-SPION (100 µg/mL) in 1640 medium for 6 h to permit cellular uptake or binding. The magnetic signals of all the groups were measured before and after 30 s of magnetization. Given that SPIONs are known to exhibit rapid post magnetization decay, an additional SPION+4T1 co-culture group (3.0 × 10⁶ cells with 100 µg/mL SPION, 6 h incubation) was included under identical conditions to enable direct comparison with F-SPION.

To investigate the effect of incubation time on F-SPION-mediated magnetic signal enhancement, 4T1 cells (3.0×10⁶) were co-incubated with 3 mL of 1640 medium containing F-SPION (100 µg/mL) for 0, 2, 4, and 6 h (n = 5). Magnetic signals were measured before magnetization and at 5, 30, and 60 min after 30 s of magnetization. To evaluate nanoparticle-cell interactions over time, Prussian blue staining and transmission electron microscopy were performed at the same time points to assess F-SPION binding and intracellular distribution. For TEM, samples were fixed in 2.5% glutaraldehyde, dehydrated, embedded in resin, sectioned, and examined under an electron microscope. In addition, cells collected under the same conditions (n = 5 per time point) were analyzed by ICP‒MS to quantitatively determine the cellular uptake of F-SPION at different incubation times.

To investigate the correlation between tumor cell number and magnetic signal intensity, 4T1 cells at quantities of 1 × 10⁵, 2 × 10⁵, 3 × 10⁵, 4 × 10⁵, and 5 × 10⁵ were incubated with 3 mL of 1640 medium containing F-SPION (100 µg/mL) for 6 h (n = 5). After incubation, the cells were washed three times with PBS to remove unbound nanoparticles, magnetized for 30 s using a rubidium magnet, and subsequently subjected to magnetic signal measurement. Linear regression analysis was performed to assess the relationship between cell number and signal intensity.

### Evaluating early chemotherapy-induced tumor cell damage and its relationship with magnetic signal intensity

To investigate whether magnetic signal intensity reflects early tumor cell damage induced by chemotherapy, 4T1 cells (3.0 × 10⁶) were divided into three groups (n = 5): a control group and two experimental groups that were incubated with DOX (0.028 µg/mL) for 24 or 48 h 43. After being incubated with DOX, the cells were washed with PBS to remove residual drug, and subsequently incubated with 3 mL of 1640 medium containing F-SPION (100 µg/mL) for 6 h. Following a second PBS wash to eliminate unbound nanoparticles, magnetic signals were recorded before and after 30 s of magnetization. To eliminate potential bias due to cell numbers, cells from each group were re-seeded at equal density (2.0 × 10⁶; n = 5) after they were incubated with DOX and washed with PBS. These re-seeded cells were then subjected to the same F-SPION incubation and magnetic signal measurement procedures as described above. Moreover, these cells were collected and analyzed by ICP-MS to quantify the uptake of nanoparticles by tumor cells under equal cell numbers and different durations of DOX treatment.

### Evaluation of signal amplification of F-SPION in tumor tissues

To determine the peak time of the magnetic signal in tumor tissue, unilateral tumor-bearing mice (n = 5 per time point) received F-SPION (2.5 mg/kg) via tail vein injection, and tumor signals were measured after 30 s of magnetization at 15, 30, 45, 60, 75, 90, 105, 120, 240, 360, and 720 min post-injection. To assess the time-dependent distribution of magnetic signals in major organs, the mice were divided into an experimental group (F-SPION) and a control group (saline). The experimental group was euthanized at 30, 60, and 90 min (n = 5 per time point) for collection of the heart, liver, spleen, lungs, and kidneys, followed by signal measurement after 30 s of magnetization. The control group was euthanized without F-SPION administration, and the organs were collected and processed using the same procedure.

To verify the ability of F-SPION to enhance the contrast between tumor tissue and surrounding normal tissue, magnetic signals from tumor and contralateral normal tissues were measured before and after 30 s of magnetization prior to F-SPION injection. The mice then received a tail vein injection of F-SPION (2.5 mg/kg). 30 min post-injection, signals were measured again before and after 30 s of magnetization. Additional measurements at 30 and 60 min post-magnetization were conducted to evaluate dynamic changes in signal intensity. All measurements were performed in five replicates.

To evaluate the effect of magnetization on F-SPION distribution in tumors, bilateral tumor-bearing mice were first subjected to baseline T₂-weighted imaging (T₂WI, repetition time [TR] = 2470 ms, echo time [TE] = 60 ms, field of view [FOV] = 100 mm, slice thickness = 1 mm) using a 16-channel mouse coil. F-SPION (2.5 mg/kg) was then administered intravenously. At 30 min post-injection, one tumor was randomly selected for 30 s of magnetization. Serial T₂WI scans were subsequently acquired at 1, 2, 4, 8, 12, and 24 h post-magnetization to monitor the time-dependent differences in F-SPION distribution between magnetized and nonmagnetized tumors. Signal intensities of both tumors at different time points were further quantified using 3D Slicer and ImageJ software to provide a comparative assessment of temporal changes. At the designated endpoints, mice were euthanized by CO₂ inhalation, and the tumors were harvested for Prussian blue staining followed by quantitative analysis using ImageJ software to calculate the percentage of positively stained area, thereby evaluating F-SPION deposition.

### Evaluation of the impact of vascular status on F-SPION distribution and signal enhancement

To evaluate the effects of vascular status on F-SPION distribution and signal enhancement, excisional wound model mice were divided into control, congested, and inflammation groups (n = 5). Congestion was induced by local treatment with 75% ethanol to promote vascular dilation, and inflammation was induced via intraperitoneal injection of LPS (20 mg/kg). F-SPION (2.5 mg/kg) was injected via the tail vein. Magnetic signals were measured 30 min post-injection, both before and after 30 s of magnetization, with additional measurements at 30 and 60 min post-magnetization to track dynamic changes. After signal acquisition, the mice were euthanized, and wound tissues were collected for histological analysis, including hematoxylin and eosin (H&E) staining, CD31 immunofluorescence, and Prussian blue staining. CD31 immunofluorescence was used to visualize changes in vascular morphology. H&E staining was performed to assess vascular architecture, tissue edema, and inflammatory cell infiltration. Prussian blue staining was used to evaluate F-SPION deposition. Given that differences in tissue nanoparticle distribution may largely arise from variations in cellular uptake capacity, we designed a complementary *in vitro* experiment, in which 4T1 cells and HUVECs were seeded in 6-well plates at appropriate densities, co-incubated with F-SPION (100 µg/mL) for 6 h, washed with PBS to remove unbound nanoparticles, fixed with paraformaldehyde, and subjected to Prussian blue staining to visualize intracellular iron uptake.

### Assessment of magnetic signal dynamics as a tumor treatment indicator

To investigate whether magnetic signal dynamics reflect the therapeutic response in tumors, unilateral tumor-bearing mice were randomly divided into a control group and an experimental group (n = 5). The experimental group received doxorubicin (2 mg/kg) every four days for a total of six doses, whereas the control group received saline. On Days 1, 7, 14, and 21, both groups received F-SPION (2.5 mg/kg) via tail vein injection. Thirty minutes later, one tumor was magnetized for 30 s, and magnetic signals were measured at 5, 30, and 60 min; tumor volumes were also recorded.

At each time point (Days 1, 7, 14, and 21), the mice in the experimental group were euthanized, and the tumors were collected for histological analysis, including H&E staining to assess necrosis, TUNEL staining to evaluate apoptosis, and Prussian blue staining to determine F-SPION deposition. The control group mice were euthanized on Day 21 for parallel histological evaluation. For TUNEL staining, images were acquired under a fluorescence microscope, the percentage of TUNEL-positive cells was quantified using ImageJ software, and the ratio of fluorescein isothiocyanate (FITC)-positive nuclei to 4′,6-diamidino-2-phenylindole (DAPI)-stained nuclei was calculated.

### Tissue and organ toxicity

At the end of the experiment, important organs (such as the heart, liver, spleen, lungs, and kidneys) were collected, and tissue sections were prepared for histological evaluation through H&E staining. Healthy untreated mice (n = 3) served as the control group.

### Hemolysis assay

Blood was collected from the orbital venous plexus of healthy mice to prepare a 2% red blood cell (RBC) suspension. F-SPION was added at concentrations of 12.5, 25, 50, 100, and 200 µg/mL, with physiological saline used as a negative control. After being incubated at 37 °C for 3 h, the samples were centrifuged, and the absorbance of the supernatant at 450 nm was measured to assess hemolysis. Each concentration was tested in triplicate (n = 3), and the experiment was repeated three times independently.

### Blood biochemical analysis

Healthy mice were divided into control and experimental groups (n = 3). The experimental group received F-SPION (2.5 mg/kg) via tail vein injection, whereas the control group received saline. Blood samples were collected on Days 1, 7, and 14 post-injection for complete blood count analysis.

### Statistical analysis

All the statistical analyses were performed using GraphPad Prism 10 (GraphPad Software, San Diego, CA, USA). For normally distributed data, paired-sample t-tests and one-way analysis of variance (ANOVA) were used to compare differences between groups. *P* < 0.05 was considered to indicate statistical significance.

## Results

### Characterization of F-SPION

**Figure 1:**
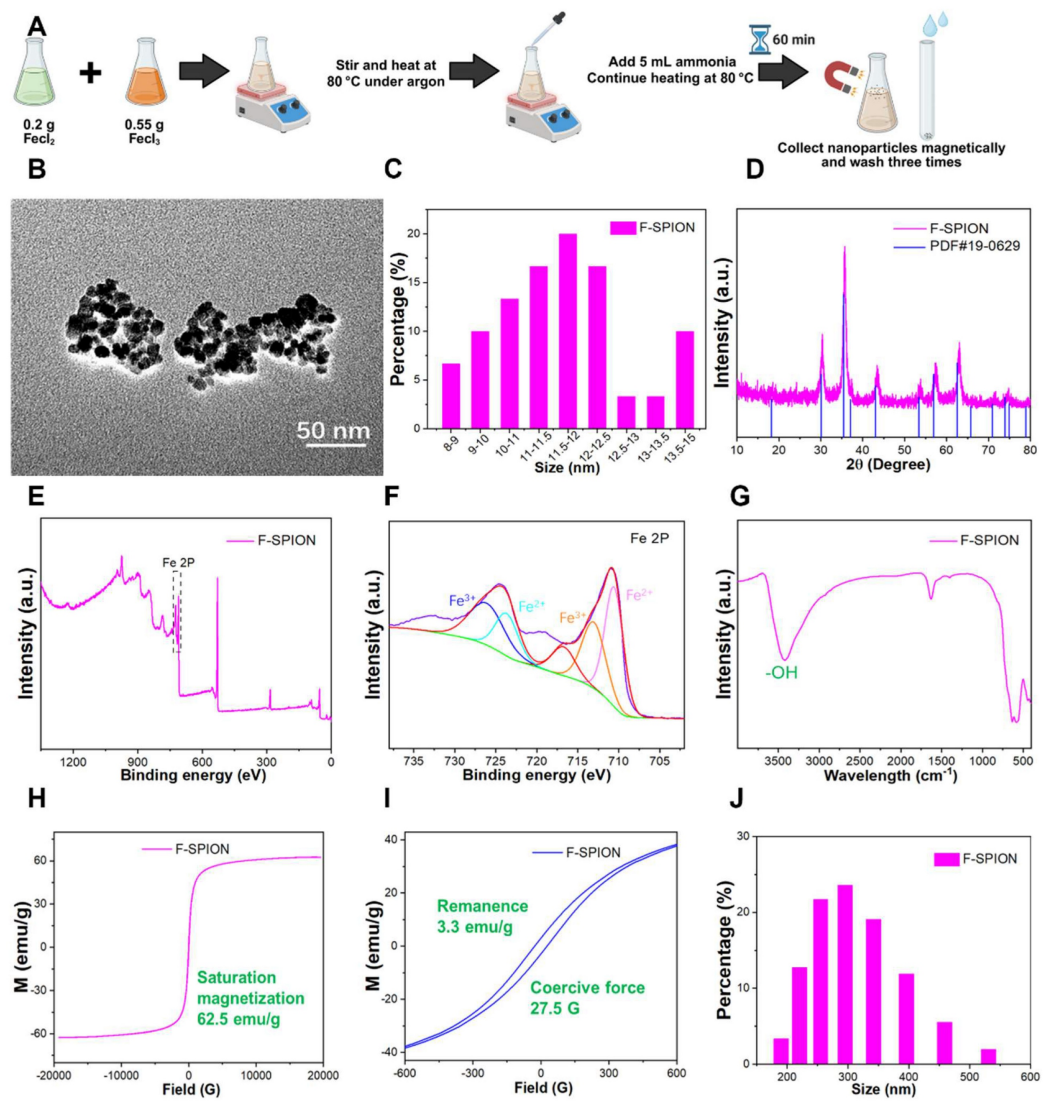
** Characterization of F-SPION.** A) Schematic illustration of F-SPION synthesis. B) TEM image of F-SPION. C) Size distribution of F-SPION determined by TEM analysis. D) XRD pattern of F-SPION. E) XPS survey spectrum of F-SPION. F) High-resolution Fe 2p XPS spectrum. G) FTIR spectrum of F-SPION. H) Hysteresis loop of F-SPION under a high magnetic field. I) Hysteresis loop of F-SPION under a low magnetic field. J) Hydrodynamic size distribution of F-SPION.

The synthesis process of F-SPION is illustrated in Figure 1A. TEM images (Figure 1B) showed nearly spherical nanoparticles with an average size of 12 ± 1.5 nm (n = 200) and good uniformity, and a higher-resolution TEM image is provided in Figure S1 to further illustrate the particle morphology and dispersion. The corresponding size distributions are presented in Figure 1C. The XRD pattern (Figure 1D) confirmed the high crystallinity of F-SPION, which is consistent with the standard PDF card 19-0629. XPS analysis (Figure 1E-F) displayed the coexistence of Fe²⁺ and Fe³⁺, confirming the mixed-valence state of F-SPION. The FTIR spectra (Figure 1G) revealed a broad absorption band at 3420.6 cm⁻¹, corresponding to -OH groups on the nanoparticle surface.

The magnetic properties measured by VSM (Figure 1H-I) showed a saturation magnetization of 62.5 emu/g, a remanence of 3.3 emu/g, and a coercive force of 27.5 G, indicating superparamagnetic behavior with weak ferromagnetic characteristics. Such weak remanence may help sustain short-term magnetic signals while minimizing background interference. The hydrodynamic size distribution measured by DLS (Figure 1J) had an average diameter of 308 ± 75 nm with a PDI of 0.323, reflecting moderate dispersion stability in aqueous solution.

### Magnetization-induced signal enhancement

**Figure 2:**
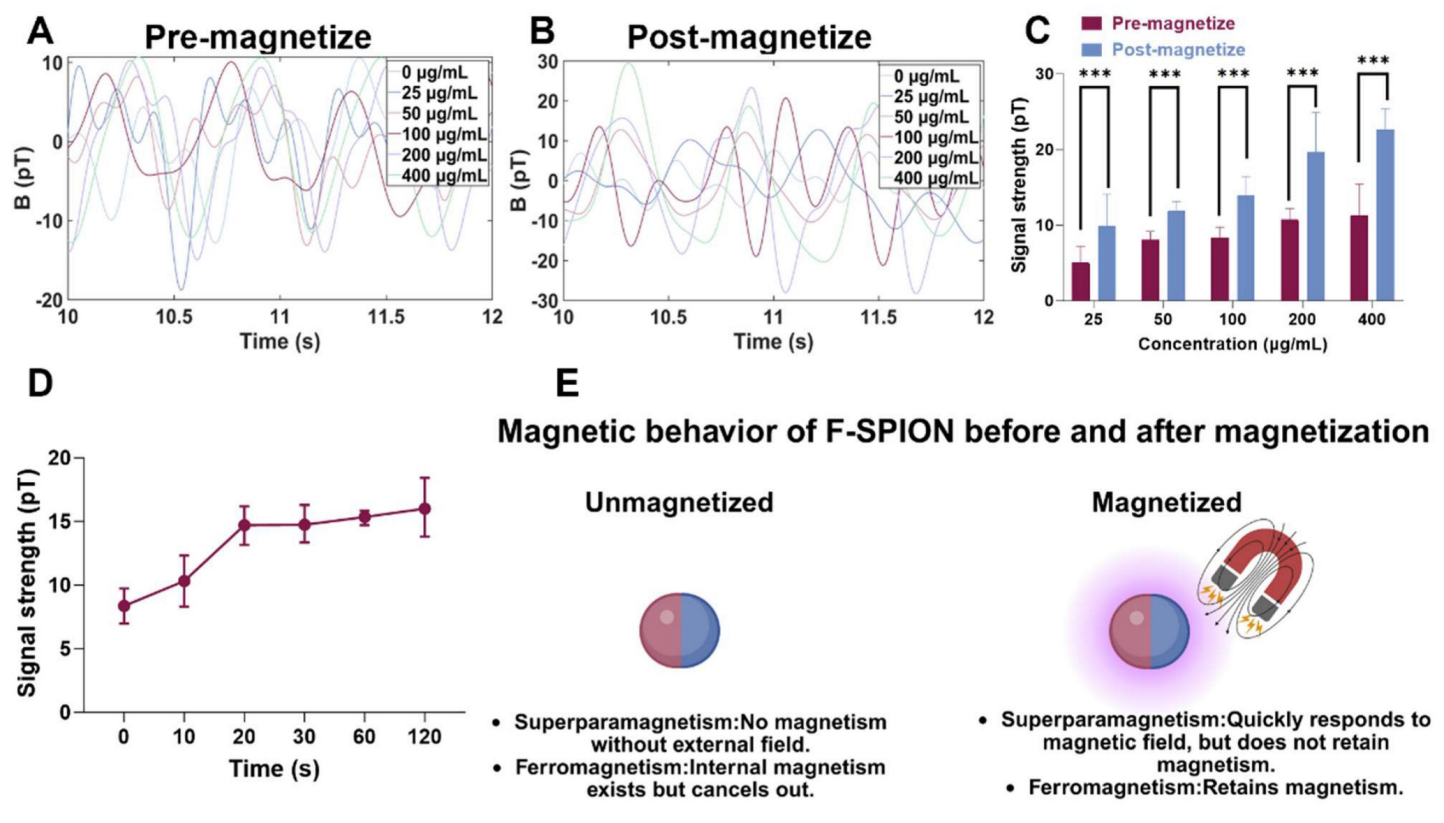
** Magnetic behavior of F-SPION.** A) Magnetic signals of F-SPION at different concentrations before magnetization. B) Magnetic signals of F-SPION at different concentrations after 30 s of magnetization. C) Comparison of F-SPION signals before and after 30 s of magnetization (n = 5). D) Magnetic signals of F-SPION at 100 µg/mL under different magnetization durations (n = 5). E) Schematic illustration of F-SPION magnetic behavior before and after magnetization. The data are presented as the mean ± standard deviation. Statistical significance was analyzed by paired t-test; ***P < 0.001.

To investigate the response of F-SPION to external magnetic fields and to identify the factors influencing changes in signal intensity, we evaluated two variables: material concentration and magnetization time. The signal intensities of F-SPION at different concentrations were measured before and after 30 s of magnetization (Figure 2A-B), while the effect of the duration of magnetization was assessed at a fixed concentration of 100 µg/mL (Figure S2). As shown in Figure 2A, F-SPION at concentrations ranging from 25 to 400 µg/mL exhibited low and comparable signal fluctuations before magnetization, with values between 5.06 ± 2.11 pT and 11.34 ± 4.10 pT. This suggests that unmagnetized F-SPION does not generate appreciable background noise, even at high concentrations. After 30 s of magnetization, the signal intensities significantly increased across all groups, as illustrated by the post-magnetization time-domain curves (Figure 2B), with values increasing from 9.89 ± 4.22 pT to 22.64 ± 2.72 pT. This concentration-dependent signal enhancement was further validated by the corresponding statistical analysis (Figure 2C). Paired-sample t-tests indicated that, for each concentration, the signal intensities after magnetization were significantly higher than those before magnetization (*P* < 0.001). These results demonstrate that F-SPION effectively amplifies magnetic signals upon exposure to external magnetic fields, confirming its excellent magnetic responsiveness.

Furthermore, to assess the impact of the duration of magnetization, F-SPION at a fixed concentration of 100 µg/mL was subjected to varying magnetization times. As shown in Figure 2D, the signal intensity progressively increased with longer magnetization duration, and a continued upward trend was observed even at 120 s.

These findings confirm that the signal enhancement of F-SPION is magnetization-dependent and positively correlated with both the duration of magnetization and the material concentration, which is consistent with the magnetic behavior of F-SPION, as summarized schematically in Figure 2E.

### Cell-nanoparticle interaction in magnetic signal enhancement

The signal enhancement induced by F-SPION is mediated by magnetic relaxation processes, which are influenced by their interaction with tumor cells. To explore the role of nanoparticle-cell binding in signal amplification, magnetic signals were measured under conditions with and without full incubation of F-SPION with tumor cells. PBS was used as a negative control, and the intrinsic magnetic responsiveness of 4T1 tumor cells was also assessed.

**Figure 3:**
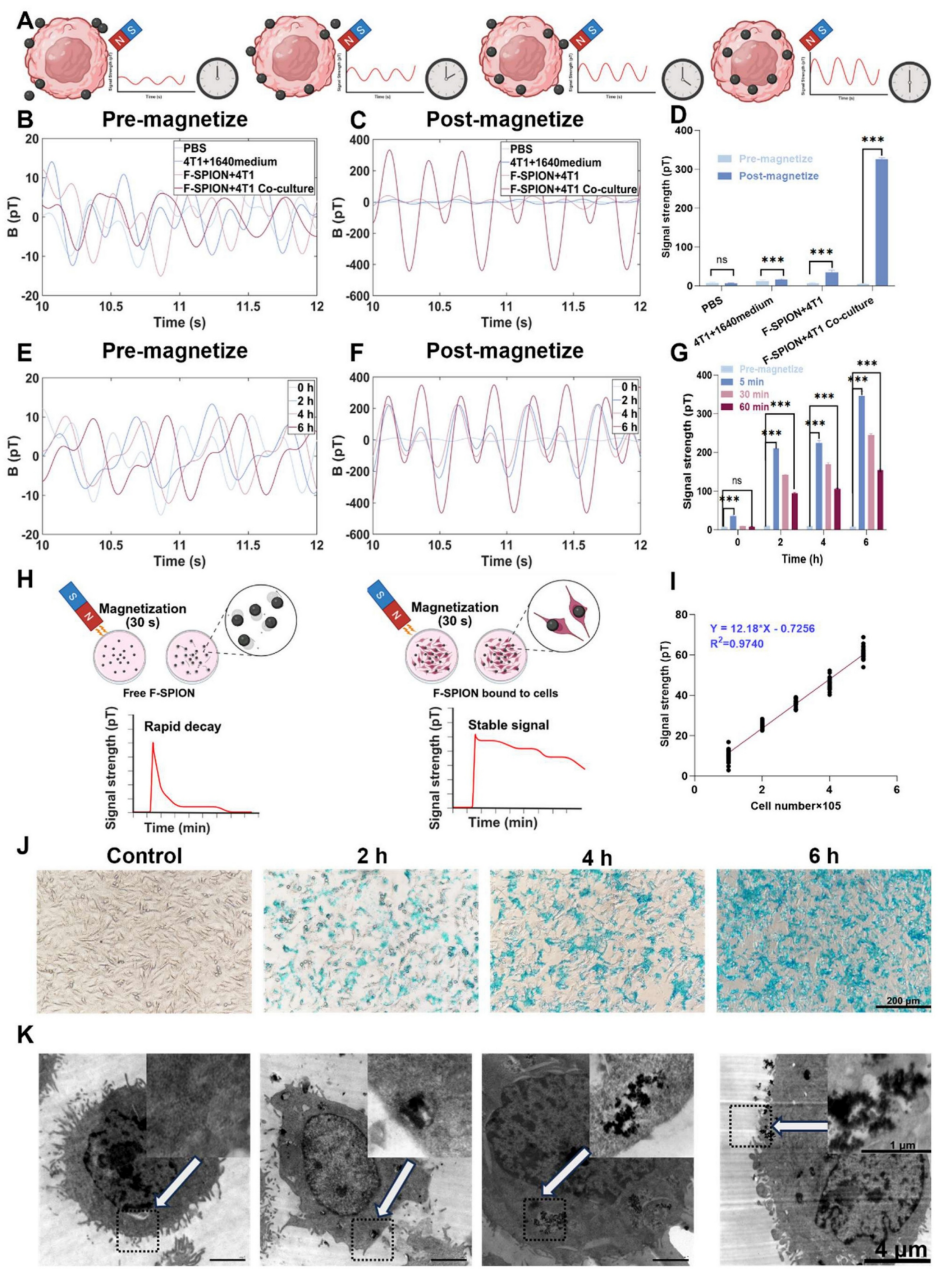
** Nanoparticle-cell interaction enhances magnetic signal.** A) Time-dependent uptake enhances post-magnetization signal intensity. B) Magnetic signals of different groups before magnetization. C) Magnetic signals of different groups after magnetization. D) Statistical comparison of signal intensities before and after magnetization (n = 5). E) Magnetic signals after different co-culture times before magnetization. F) Magnetic signals measured 5 min after 30 s of magnetization under different co-culture durations. G) Signal intensities before magnetization and at 5, 30, and 60 min after magnetization under different co-culture durations (n = 5). H) Schematic illustration comparing signal decay between free and cell-bound F-SPION. I) Linear correlation between cell number and signal intensity (R² = 0.974) (n = 5). J) Prussian blue staining shows time-dependent uptake of F-SPION by 4T1 cells. K) TEM images confirm the time-dependent uptake and intracellular localization of F-SPION. White arrows indicate the regions displayed at higher magnification (30,000×) in the inset images. The data are presented as the mean ± standard deviation. Statistical significance was analyzed by paired t-test; ns, not significant; ***P < 0.001.

As shown in Figure 3B, all groups exhibited low signal intensities under unmagnetized conditions, ranging from 5.12 ± 1.18 pT to 11.84 ± 3.50 pT. Following magnetization (Figure 3C), the signals of the co-culture group dramatically increased from 5.12 ± 1.18 pT to 326.27 ± 3.24 pT, corresponding to an approximately 6400% enhancement. In contrast, the F-SPION + 4T1 group showed a moderate increase from 6.39 ± 1.97 pT to 36.01 ± 2.87 pT, while the other groups exhibited no substantial change (Figure 3D). These results indicate that 4T1 cells alone possess limited intrinsic magnetic responsiveness, and that the presence of F-SPION is essential for signal amplification. Notably, although both the co-culture group and the F-SPION + 4T1 group contained the same amount of nanoparticles, a significant difference in signal intensity was observed between them, underscoring that signal amplification requires not only external magnetization but also sufficient nanoparticle-cell interaction. To further assess differences in signal stability, we compared the magnetic responses of the SPION+4T1 co-culture and F-SPION+4T1 co-culture groups under identical conditions. As shown in Figure S3, under identical dosing conditions, the SPION+4T1 co-culture group yielded only near-baseline signals, whereas the F-SPION+4T1 co-culture group exhibited markedly enhanced magnetic signals. These results indicate that F-SPIONs maintain relatively stable signals with slower post-magnetization decay, allowing detectable outputs, while SPIONs decay too rapidly to enable reliable monitoring. Therefore, F-SPIONs serve as more stable and practical magnetic signal enhancers for further applications.

Because these findings indicate that stable amplification requires sufficient nanoparticle-cell interaction, we next investigated how incubation duration influences signal enhancement. As shown in Figure 3E, before magnetization, the signal intensities remained relatively stable across all groups, ranging from 6.39 ± 1.97 pT to 10.89 ± 2.29 pT with increasing incubation time. After magnetization, magnetic signals were measured at 5, 30, and 60 min. At 5 min post-magnetization (Figure 3F), the peak signal intensities progressively increased, reaching 210.40 ± 16.50 pT, 224.39 ± 5.99 pT, and 344.64 ± 11.46 pT after 2, 4, and 6 h of incubation, respectively, indicating that longer incubation times enhanced the signal amplification effect. At 30 min after magnetization (Figure S4), the signal intensities were 8.75 ± 1.35 pT, 141.49 ± 1.84 pT, 167.76 ± 8.13 pT, and 244.70 ± 3.65 pT after 0, 2, 4, and 6 h of incubation, respectively. At 60 min after magnetization (Figure S5), the signals were 7.91 ± 0.87 pT, 93.88 ± 4.13 pT, 106.21 ± 2.17 pT, and 163.62 ± 2.68 pT after 0, 2, 4, and 6 h, respectively. One-way analysis of variance (Figure 3G) revealed that for samples incubated for 0 h, the magnetic signal intensity at 60 min post-magnetization was not significantly different from that before magnetization, whereas for samples incubated for 2, 4, and 6 h, the magnetic signal intensities at 60 min remained significantly higher than the corresponding pre-magnetization levels.

To further investigate the microscopic mechanisms underlying signal amplification, Prussian blue staining and transmission electron microscopy were performed to assess the intracellular distribution of F-SPION after different incubation durations. Prussian blue staining (Figure 3J) revealed a time-dependent increase in intracellular iron accumulation, with more extensive blue staining observed as the incubation time extended. Transmission electron microscopy (Figure 3K) further confirmed the progressive internalization of F-SPION, revealing the formation of electron-dense aggregates within tumor cells over time.

To quantify the relationship between incubation time and nanoparticle uptake by tumor cells, ICP-MS was employed to measure the intracellular iron content in 4T1 cells at different time points. As shown in Figure S6, the intracellular iron content in tumor cells progressively increased with increasing incubation time.

These results demonstrate that magnetic signal intensity reflects the amount of F-SPION internalized by tumor cells, with longer incubation durations leading to greater nanoparticle uptake and more pronounced signal amplification. By contrast, extracellular nanoparticles that remained unbound to cells exhibited rapid signal decay after magnetization, as shown in Figure 3H. These combined findings indicate that sustained magnetic signals after magnetization can be achieved only when F-SPION is internalized by tumor cells, highlighting the critical role of nanoparticle-cell interaction in maintaining long-term signal stability. Given this association, under a fixed incubation duration, magnetic signal intensity can potentially serve as a functional indicator of the capacity of tumor cells to internalize F-SPION.

In addition to uptake capacity, the tumor response to chemotherapy also manifests as a reduction in tumor cell number, which directly impacts the quantity of F-SPION internalized and, consequently, the magnetic signal intensity. To investigate this relationship, magnetic signals were measured at varying cell densities. As shown in Figure S7, the peak signal intensity after magnetization increased from 13.25 ± 4.28 pT at 100,000 cells to approximately 60.97 ± 2.47 pT at 500,000 cells, demonstrating a positive correlation between signal intensity and cell number. Linear regression analysis (Figure 3I) revealed a strong linear relationship, with a coefficient of determination (R²) of 0.974. These findings indicate that magnetic relaxation technology can sensitively reflect tumor burden through changes in magnetic signal intensity.

### Magnetic signals reflect early chemotherapy-induced tumor cell damage

The results of the aforementioned experiments confirmed that magnetic relaxation technology reflects changes in tumor cell quantity via signal intensity. Given that chemotherapy drugs not only induce apoptosis but also suppress tumor cell viability and nanoparticle uptake capacity 41, we next assessed whether magnetic signals can also reflect chemotherapy-induced cellular damage and impaired uptake function. Accordingly, 4T1 cells were treated with DOX for 24 or 48 h, while untreated cells served as controls. The DOX concentration used in this study was based on previously reported IC₅₀ values for 4T1 cells. Magnetic signals were measured before and after magnetization, and F-SPION uptake was evaluated by Prussian blue staining.

As shown in Figure S8, before magnetization, the signal intensities were similar across the different groups. After magnetization (Figure S9), the signal intensity of the control group was 399.32 ± 6.33 pT, while the group treated with DOX for 24 h had a signal intensity of 274.93 ± 7.03 pT, and the group treated with DOX for 48 h had a signal intensity of 68.83 ± 3.93 pT, which was less than 50% of the control group. Importantly, although the DOX concentration was selected based on the reported IC₅₀ for 4T1 cells, the signal intensity observed in the 48 h treatment group was markedly lower than 50% of the control group. This discrepancy suggests that, in addition to reducing tumor cell number, DOX may further compromise the ability of cells to internalize F-SPION or interfere with nanoparticle-cell interaction, thereby amplifying the decrease in signal intensity. As shown in Figure S10, the statistical analysis of signal intensities before and after magnetization further confirmed the trends observed in Figures S8 and S9. To validate this assumption, Prussian blue staining was performed. The results (Figure S11) revealed that as the duration of DOX treatment increased, the number of tumor cells decreased, and the phagocytosis of F-SPION by tumor cells significantly reduced.

**Figure 4:**
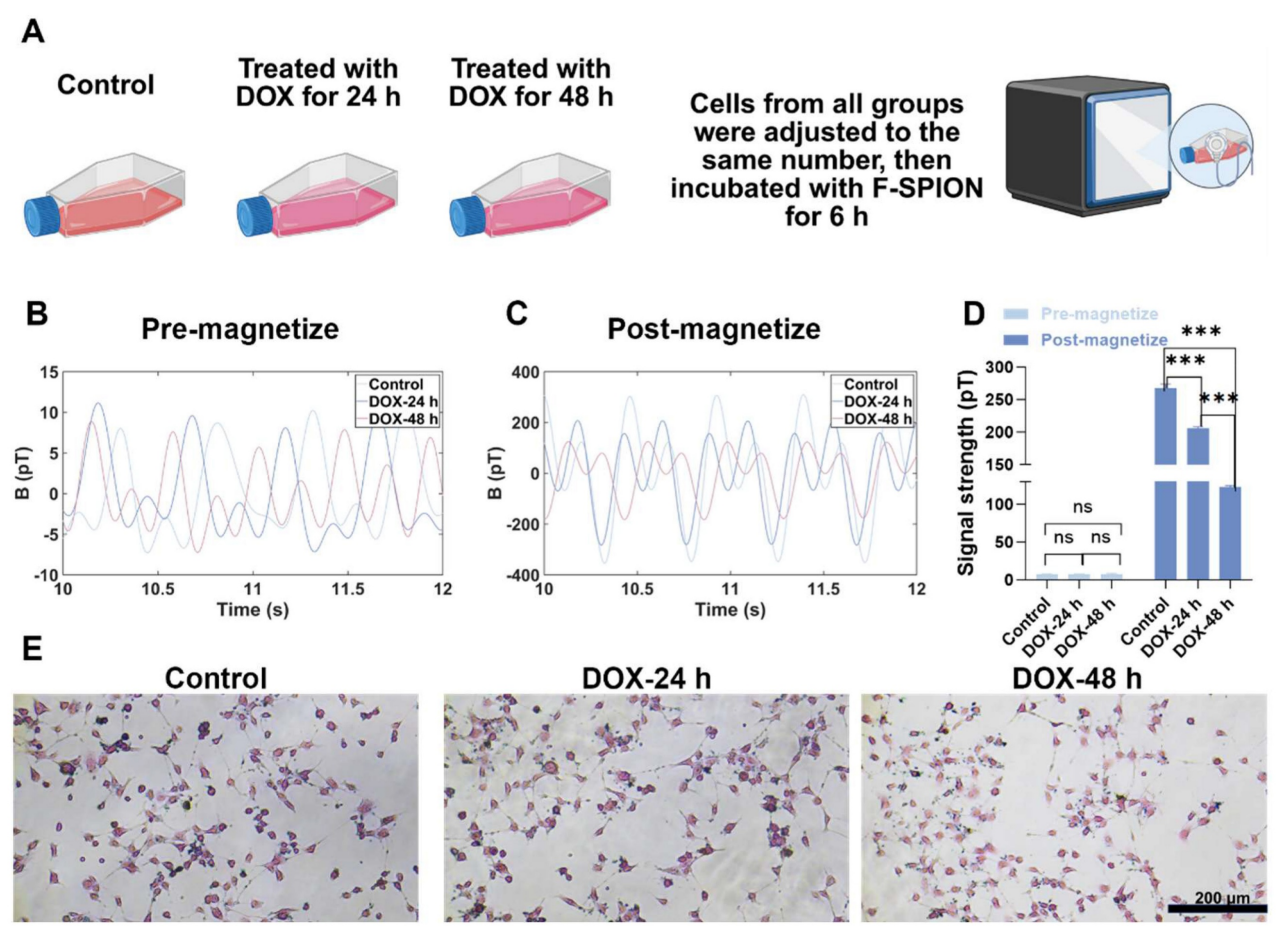
** Magnetic signals reflect early chemotherapy-induced tumor cell damage.** A) Experimental workflow. B) Magnetic signals of the control and DOX-treated groups before magnetization. C) Magnetic signals of the same groups after 30 s of magnetization. D) Statistical comparison of signal intensities before and after 30 s of magnetization (n = 5). E) Prussian blue staining shows reduced F-SPION uptake after prolonged DOX treatment. The data are presented as the mean ± standard deviation. Statistical significance was analyzed by one-way ANOVA; ns, not significant; ***P < 0.001.

To eliminate the influence of tumor cell quantity on the measurements, the magnetic signal intensity of the same number of cells from the three groups mentioned above was measured. Like in Figure S8, the magnetic signals before magnetization (Figure 4B) differed minimally across the groups. As shown in Figure 4C, the magnetic signal intensity of the control group was 267.88 ± 5.97 pT, while the intensity of the group treated with DOX for 24 h decreased to 206.02 ± 2.23 pT, and that of the group treated with DOX for 48 h further decreased to 122.74 ± 2.11 pT. Consistently, as shown in Figure 4E, Prussian blue staining revealed that F-SPION uptake by tumor cells progressively decreased with increasing duration of DOX treatment. To quantitatively assess the effects of different durations of DOX treatment on nanoparticle uptake by tumor cells, ICP-MS was used to measure the intracellular iron content in 4T1 cells. As shown in Figure S12, with increasing DOX treatment time, tumor cell nanoparticle uptake progressively decreased.

These findings demonstrate that magnetic signals can sensitively reflect both chemotherapy-induced tumor cell apoptosis and the impairment of nanoparticle uptake capacity. This dual responsiveness highlights the potential of magnetic signal-based detection as a noninvasive and comprehensive approach for monitoring treatment effects, evaluating drug efficacy, and guiding therapeutic optimization.

### Signal amplification of F-SPION in tumor tissues

In traditional imaging modalities, the imaging efficacy of contrast agents depends on their distribution within the body, especially for tumor imaging where targeting is vital. To observe the distribution and clearance patterns of F-SPION, the signal intensities of magnetized tumor tissues and major organs were measured at various time points.

**Figure 5:**
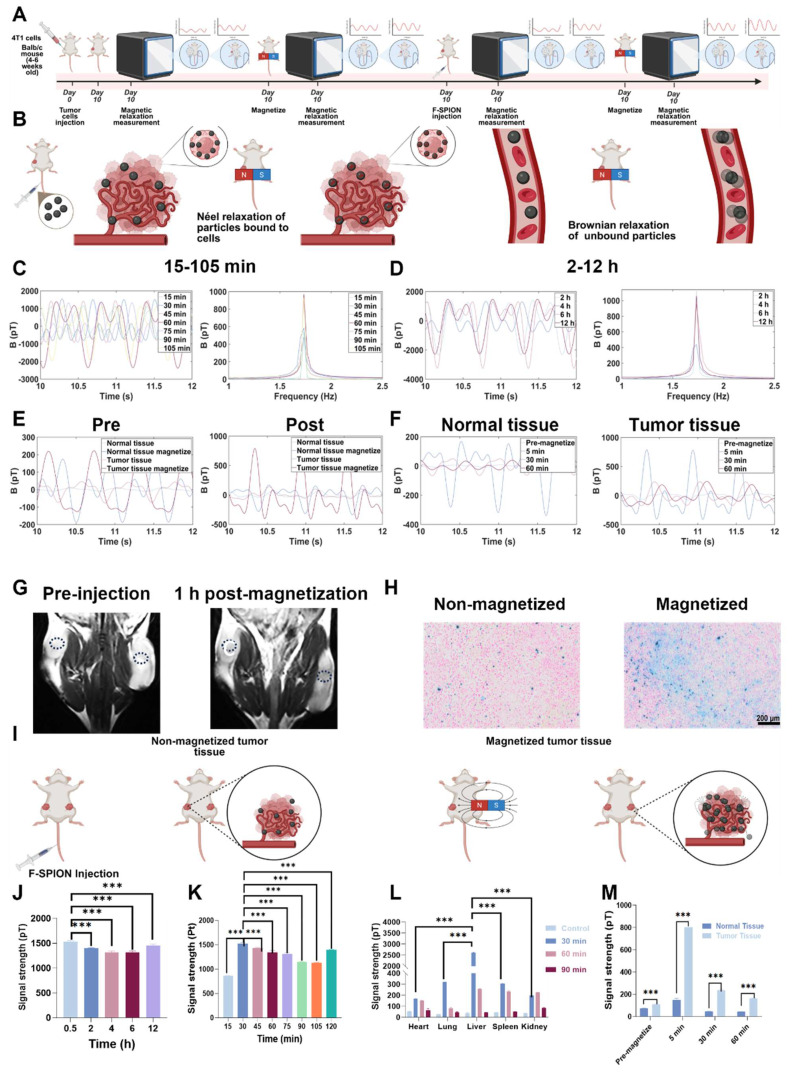
**Signal amplification of F-SPION in tumor tissues.** A) Experimental workflow. B) Relaxation mechanisms of nanoparticles *in vivo*. C) Changes in tumor signal (15-105 min post-injection). D) Changes in tumor signal (2-12 h post-injection). E) Signal intensity before and after F-SPION injection. F) Changes in signal intensity over time in normal and tumor tissues. G) MR images of tumor-bearing mice, with tumors located bilaterally in the inguinal region on the dorsal side. The average tumor volume was 216 ± 22 mm³ (n = 5). The dotted circles indicate the regions of interest used for quantifying tumor enhancement values. H) Prussian blue staining of tumor tissue. I) Schematic illustration of the F-SPION distribution with and without magnetic activation. J) Statistical comparison of tumor signal intensities (0.5-12 h post-injection) (n = 5). K) Statistical comparison of tumor signal intensities (15-120 min post-injection) (n = 5). L) Signal intensity in major organs (n = 5). M) Statistical analysis of signal intensity in tumor and normal tissues (n = 5). The data are presented as the mean ± standard deviation. Statistical significance was analyzed by one-way ANOVA; ***P < 0.001.

Magnetic signal measurements (Figures 5C-D) showed that the signal intensity in tumor tissue reached 868.02 ± 7.71 pT at 15 min post-injection. The signal peaked at 1528.54 ± 23.34 pT at 30 min, and the corresponding statistical analyses are presented in Figures 5J-K. Although a gradual decline was observed thereafter, the signal remained at a relatively high level. Specifically, signal intensities at 45, 60, 75, 90, 105, and 120 min post-injection were 1434.40 ± 14.47 pT, 1338.68 ± 59.31 pT, 1314.35 ± 25.84 pT, 1143.99 ± 17.29 pT, 1132.18 ± 2.71 pT, and 1395.79 ± 13.70 pT, respectively. At 4 h post-injection, the signal remained at 1313.30 ± 32.42 pT, and continued to be elevated at 6 and 12 h, with values of 1313.59 ± 32.70 pT and 1449.66 ± 30.79 pT, respectively. These results demonstrate that following tail vein injection, F-SPION rapidly accumulate in tumor tissue and are retained at that level for extended periods. The sustained high signal levels over long time frames indicate that F-SPION-based magnetic relaxation measurements using SERF magnetometers offer a relatively broad detection window, which may facilitate translation to clinical applications.

As shown in Figure S13, magnetic signals from major organs in mice without F-SPION injection remained low and stable after 30 s of magnetization. At 30 min post-injection, the signal intensity of the liver was significantly higher than that of other organs, indicating that F-SPION was rapidly recognized by the reticuloendothelial system of the liver and cleared by this organ (Figure S14). Consistently, statistical analysis confirmed that the liver signal was the strongest, as shown in Figure 5L. The spleen also showed elevated signal intensity, suggesting some degree of F-SPION clearance. The heart retained a relatively high signal, reflecting the presence of circulating nanoparticles. The kidney exhibited increased signal intensity, indicating F-SPION entry and potential renal excretion. By 60 min (Figure S15), both liver and spleen signal intensities decreased significantly, suggesting that F-SPION metabolism or degradation occurred within these organs. The heart signal also decreased, indicating a reduction in circulating F-SPION. The lung signals were the lowest, indicating minimal retention. The kidney signal further increased, suggesting that F-SPION may be excreted via the kidneys. At 90 min post-injection (Figure S16), the signal intensities in all organs declined, indicating gradual metabolism and clearance of F-SPION. In contrast, tumor tissue retained F-SPION for a longer period, enhancing the contrast between tumor and normal tissue magnetic signals.

To further assess the impact of F-SPION on tumor tissue signals, the signal intensities of tumor tissues before and after F-SPION injection were measured, with contralateral normal tissues serving as controls. As shown in Figure 5E, before F-SPION injection, magnetization increased the signal intensity in tumor tissues from 44.26 ± 1.27 pT to 223.03 ± 3.79 pT, whereas that in control tissues increased from 9.5 ± 1.26 pT to 163.42 ± 3.95 pT. After F-SPION injection, the signal intensity in tumor tissues increased from 108.48 ± 5.29 pT to 802.70 ± 60.81 pT, whereas in control tissues, the signal intensity increased from 72.60 ± 2.88 pT to 149.25 ± 16.23 pT. These findings clearly demonstrate that F-SPION significantly enhances the signal contrast between tumor and normal tissues.

As shown in Figure 5F, at 60 min post-magnetization, the signal intensity of normal tissues decreased to 42.75 ± 1.49 pT, indicating a return to baseline levels, whereas tumor tissues maintained a high signal intensity of 163.57 ± 3.20 pT. This sustained high contrast between tumor and normal tissues, underscores the potential of F-SPION for providing strong and lasting contrast in tumor imaging.

To investigate the impact of magnetization on F-SPION distribution in tumors, bilateral tumor-bearing mice were subjected to T₂-weighted imaging. As shown in Figure 5G, MRI revealed a decrease in T₂ signal intensity in tumor tissues following F-SPION injection, with a more pronounced reduction observed on the magnetized side (right). The magnetic resonance images in Figure S17 and the signal intensity measurements in Figure S18 show that, at different time points post-magnetization, the signal intensity in the magnetized side is lower than that in the non-magnetized side, suggesting that magnetization promotes the local aggregation of F-SPION, leading to enhanced magnetic susceptibility effects and T₂ signal attenuation. Consistent with these findings, Prussian blue staining (Figure 5H) demonstrated greater F-SPION accumulation in magnetized tumors than in their non-magnetized counterparts. Quantitative analysis (Figure S19) further confirmed this observation, showing that the percentage of Prussian blue-positive area in magnetized tumors was nearly 100%, whereas non-magnetized tumors displayed only minimal staining. These findings further support that magnetic activation facilitates the targeted aggregation and retention of F-SPION within tumor tissues.

### Impact of vascular status on F-SPION distribution and signal enhancement

Previous studies have verified the key role of the uptake of F-SPION by tumor tissue in signal enhancement; however, nanoparticles must travel through blood vessels to reach tumor tissue. To assess the impact of vascular status, signals from congested and inflamed wound tissues were measured, with ordinary wounds used as the control.

**Figure 6:**
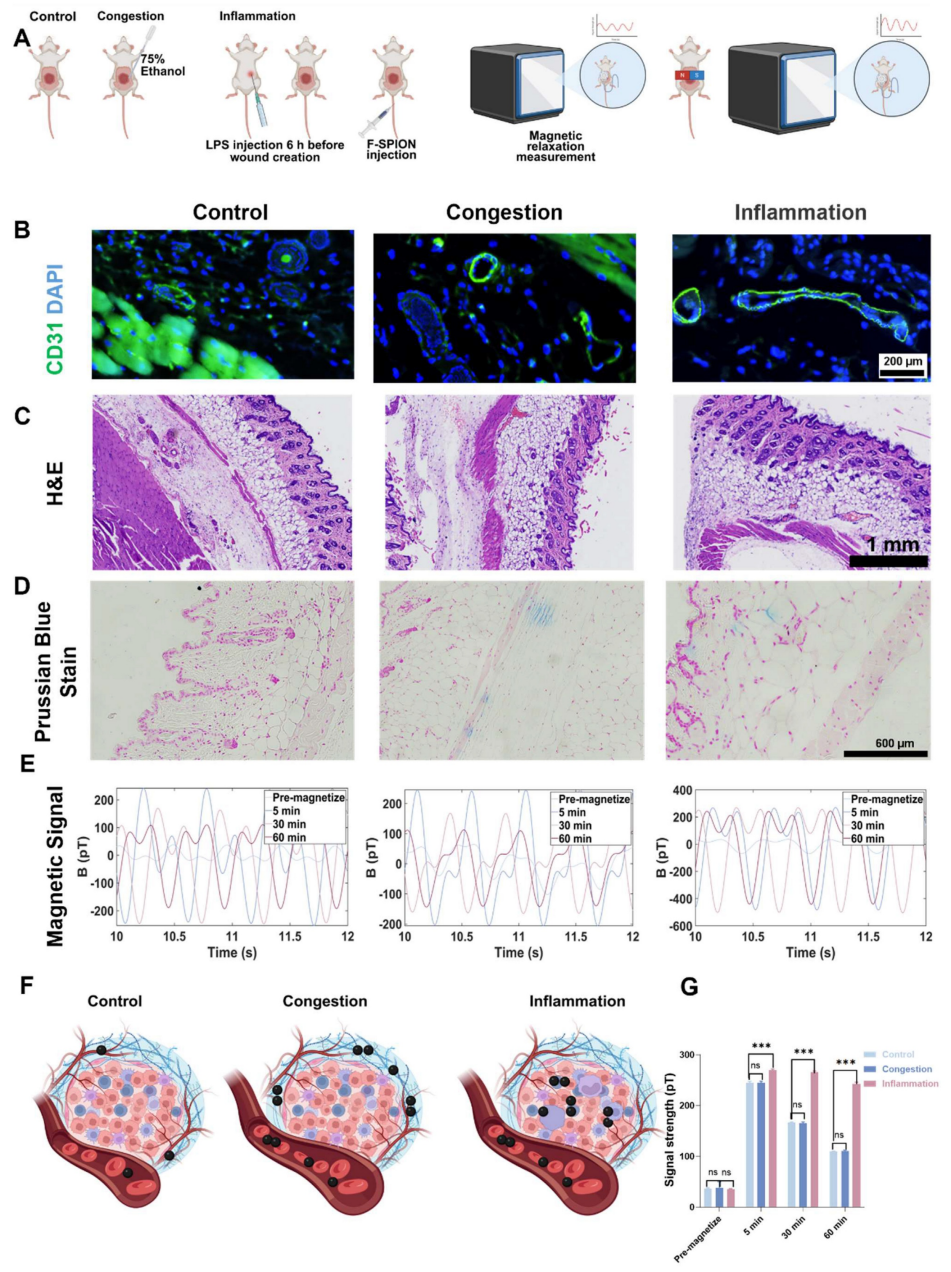
** Impact of vascular status on F-SPION distribution and signal enhancement.** A) Experimental workflow. B) Immunofluorescence staining of CD31 (green) and DAPI (blue) in the control, congestion, and inflammation groups. C) H&E staining of all groups. D) Prussian blue staining of all groups. E) Changes in signal intensity over time in all groups. F) Schematic illustration of F-SPION distribution under different vascular conditions. G) Statistical comparison of magnetic signal intensities among all groups (n = 5). The data are presented as the mean ± standard deviation. Statistical significance was analyzed by one-way ANOVA; ns, not significant; ***P<0.001.

In the control group, CD31 staining of the wound tissue samples revealed small and well-defined blood vessel profiles (Figure 6B). In contrast, the congestion and inflammation groups exhibited significantly enlarged vascular lumens, with more pronounced dilation. The H&E staining results shown in Figure 6C revealed that the control group had a compact tissue structure with clear skin layers, and no signs of vascular dilation or inflammatory cell infiltration. Compared with those in the control group, the blood vessels in the congestion group were noticeably dilated, and the connective tissue gaps were wider. The inflammation group also showed vascular dilation and tissue loosening, with extensive inflammatory cell infiltration in the stroma, indicating that the local tissue was in an activated inflammatory state.

Prussian blue staining (Figure 6D) showed minimal blue staining signals in the control group wound tissue, suggesting limited F-SPION deposition. In the congestion group, blue stained particles were distributed primarily in the connective tissue gaps. In the inflammation group, blue-stained particles were localized mainly inside the cells, indicating that F-SPION was internalized by the cells.

Magnetic signal measurements (Figure 6E) showed that prior to magnetization, the signal intensities of the wound tissues from the control, congestion, and inflammation groups were 36.07 ± 11.94 pT, 38.10 ± 12.26 pT, and 35.21 ± 1.38 pT, respectively. One-way ANOVA (Figure 6G) confirmed that there were no statistically significant differences in the magnetic signal intensity of the three groups before magnetization. After magnetization, the inflammation group showed significantly greater magnetic signal intensities at 5, 30, and 60 min (269.48 ± 2.62, 265.88 ± 2.20, and 242.80 ± 3.24 pT, respectively) compared with the control (245.20 ± 2.72, 166.18 ± 1.62, and 109.56 ± 1.04 pT) and congestion groups (245.00 ± 3.00, 166.05 ± 1.63, and 110.22 ± 2.29 pT; one-way ANOVA, *P* < 0.001). By contrast, there were no significant differences in magnetic signal intensity between the control and congestion groups at any time point (*P* > 0.05).

These findings suggest that effective signal enhancement requires cellular uptake of F-SPIONs followed by activation under an external magnetic field. Nanoparticles that are not internalized do not generate appreciable background signals after magnetization. Consistently, Prussian blue staining (Figure S20) demonstrated substantially greater F-SPION uptake in tumor cells than in endothelial cells, providing a mechanistic explanation for the stronger magnetic signals observed in tumor tissues.

### Magnetic signal as a tumor treatment indicator

**Figure 7:**
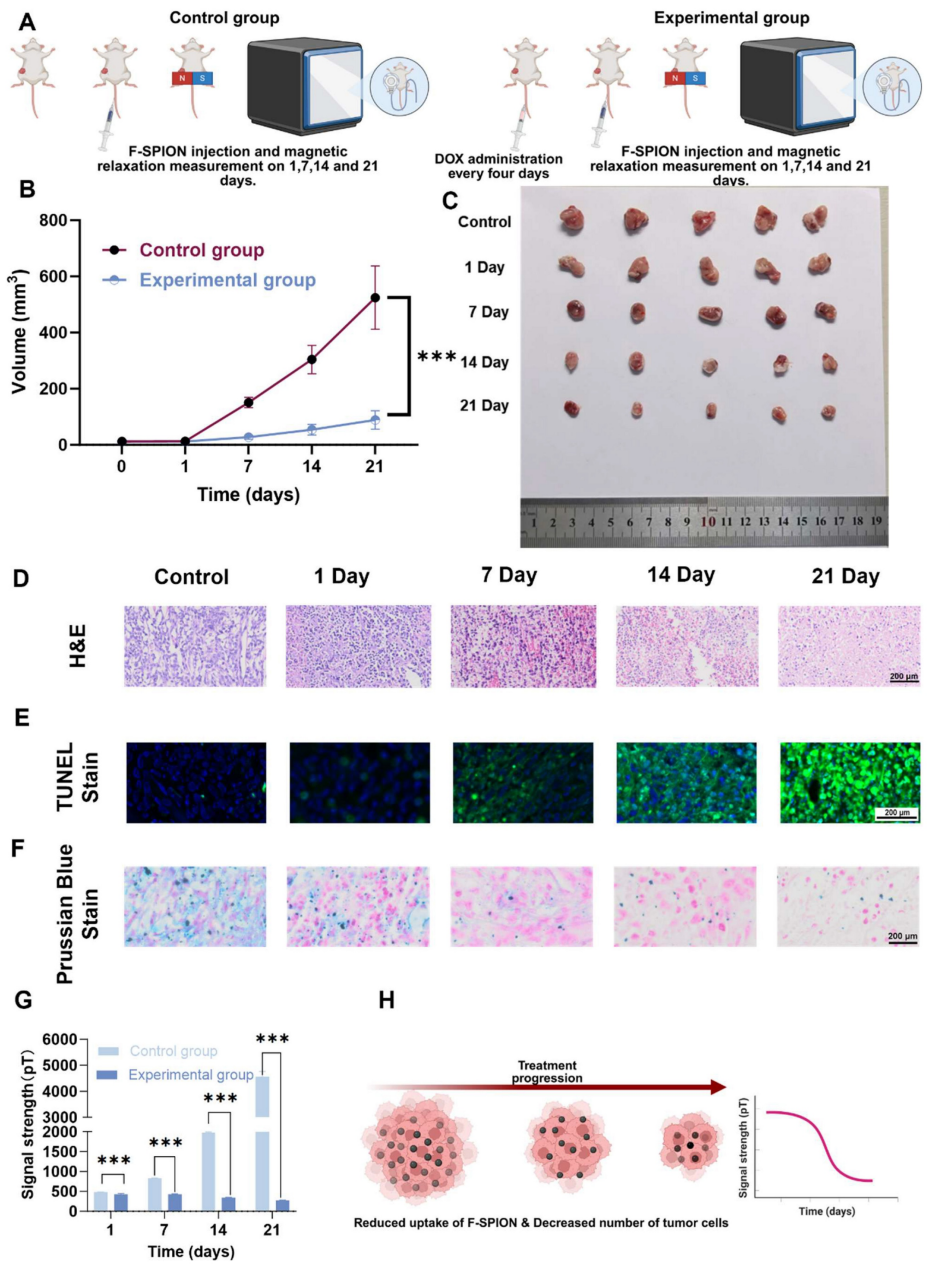
** Magnetic signal as a tumor treatment indicator.** A) Experimental workflow. B) Tumor growth curves of the control and experimental groups at different time points (n = 5). C) Representative images of excised tumors collected at Day 21 after different treatment durations (1, 7, 14, and 21 days). D) H&E staining of tumor tissues. E) TUNEL staining showing apoptosis in tumor sections. F) Prussian blue staining of tumor tissues. G) Statistical analysis of magnetic signal intensity differences across all time points (n = 5). H) Schematic illustration showing how treatment-induced tumor shrinkage and reduced F-SPION uptake led to decreased magnetic signals. The data are presented as the mean ± standard deviation. Statistical significance was analyzed by one-way ANOVA, ***P < 0.001.

To evaluate the effectiveness of SERF combined with F-SPION for monitoring therapeutic response, tumor-bearing mice were divided into control and treatment groups. As shown in Figure 7B, the tumor volume in the control group increased rapidly, whereas that in the treatment group grew slowly. Ex vivo tumor images (Figure 7C) further demonstrated the progressive shrinkage of tumors in the treatment group with prolonged therapy. Histological analyses supported these findings: H&E and TUNEL staining (Figures 7D-E) revealed increasing necrosis and apoptosis in tumor tissues over time, while quantitative analysis of TUNEL staining (Figure S21) confirmed a gradual increase in apoptotic cell ratios. Consistently, Prussian blue staining (Figure 7F) indicated a time-dependent decrease in F-SPION uptake by tumor cells, reflecting reduced tumor viability.

Magnetic signal measurements corroborated these pathological changes (Figure S22 and Figure 7G). On Day 1, the magnetic signal intensities of tumor tissues at 5 min post-magnetization were comparable between the control and treatment groups (481.41 ± 3.82 pT vs. 425.32 ± 24.40 pT). By Day 7, the signal intensity of the control group significantly increased (830.72 ± 5.90 pT), whereas that of the treatment group remained stable (425.41 ± 14.37 pT). Paired t-tests revealed significant differences between Day 1 and Day 7 in the control group (*P* < 0.001), but not in the treatment group (*P* > 0.05). At later time points, the signal intensities in the control group continued to rise (1978.70 ± 14.36 pT on Day 14; 4562.61 ± 196.84 pT on Day 21), while those in the treatment group remained low (346.62 ± 5.15 pT and 279.04 ± 3.84 pT, respectively). Notably, from Day 7 onward, the magnetic signals of the treatment group plateaued, in contrast to the steep increases observed in the control group.

Together, these results demonstrate that SERF magnetometry combined with F-SPION detection can sensitively capture therapeutic responses as early as Day 7. The distinct signal trajectories between groups provide an early observation window for efficacy evaluation and confirm the feasibility of this approach as a reliable indicator for treatment monitoring.

### Biosafety

**Figure 8:**
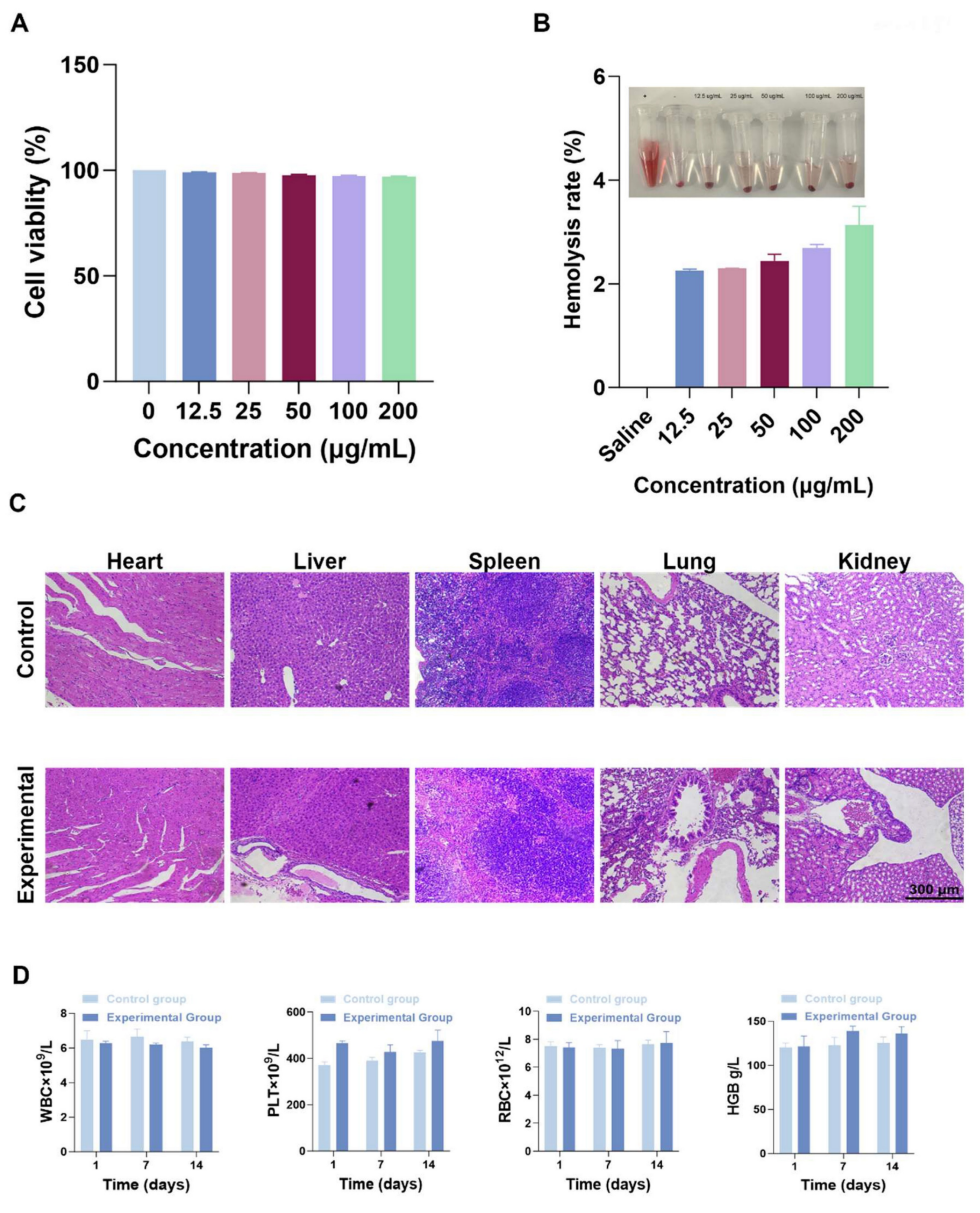
** Biosafety.** A) The cell viability of 4T1 cells remains high (near 100%) at concentrations of 12.5-200 µg/mL (n = 5). B) Results of the hemolysis assay of F-SPION (n = 5). C) Histological staining of major tissues and organs. D) Blood biochemical analysis (n = 5). The data are presented as the mean ± standard deviation.

The biological safety of F-SPION was evaluated through cytotoxicity, blood compatibility, tissue pathology, and blood biochemistry. As shown in Figure 8A, F-SPION had a minimal effect on cell viability, with survival rates ranging from 97% to 100% across concentrations of 12.5 to 200 µg/mL. The hemolysis rates slightly increased with increasing F-SPION concentration but remained very low, indicating good blood compatibility (Figure 8B). The H&E staining results shown in Figure 8C indicate that the major organs in both the experimental and control groups exhibited normal tissue architecture and orderly cellular arrangements. As shown in Figure 8D, F-SPION (2.5 mg/kg) administered via tail vein injection did not cause significant alterations in the measured hematological parameters over the 14-day observation period.

## Discussion

F-SPION enhances signals by binding to tumor cells and being activated by an external magnetic field. In the study by Soukup et al., internalized SPION particles primarily exhibited Néel relaxation due to restricted movement 44, while Hathaway et al. reported that freely moving SPION particles primarily undergo Brownian relaxation, with a relaxation time of 2 ms 45. The rapid decay of these nanoparticles ensures that they are fully attenuated at the start, preventing interference with the results. The Néel relaxation of internalized particles lasts longer, typically in the second range, but is still too short for clinical requirements of signal stability and detectability. Although ferromagnetic materials retain residual magnetism after field removal, this increases background noise and reduces contrast. To maintain a good signal-to-noise ratio, Enpuku et al. modified ferromagnetic nanoparticles with antibodies, enabling specific binding to target antigens 39. However, antigen-antibody specificity limits their use in other tumors. Moreover, test samples often contain both static magnetic fields (from magnetic substances) and dynamic magnetic fields (from physiological activities). The dynamic field is too small to measure directly; but even though the static field is within the range of the detector, only changing magnetic field signals can be captured. To address this, in this study, the samples were rotated to induce periodic changes in the static field, enabling detection. Unmagnetized F-SPION, whether freely moving or internalized, showed no detectable magnetism, minimizing background noise. After magnetization, uninternalized F-SPION underwent Brownian relaxation with rapid signal decay, enhancing the detection specificity. With just 30 s of magnetization, F-SPION increased the magnetic signal in tumor tissues from 108.48 ± 5.29 pT to 807.70 ± 60.81 pT, while that in normal tissues increased from 72.60 ± 2.88 pT to 149.25 ± 16.23 pT. F-SPION can respond quickly to the magnetic field and provide good contrast after magnetization. This difference persisted even after 60 min (tumor: 163.57 ± 3.20 pT; normal: 42.75 ± 1.49 pT), indicating that the signal enhancement effect remains without additional surface modification.

Early effects of chemotherapy on tumors extend beyond apoptosis and volume reduction, and include decreases in viability, phagocytic capacity, and metabolic activity—changes that traditional structural imaging often miss. Experiments confirmed a strong linear relationship between tumor cell number and signal intensity; however, treating 4T1 cells with DOX at the IC_50_ for 48 h reduced their signal to well below 50% of that in untreated cells, indicating that the signal intensity depends on more than the cell count. Prussian blue staining revealed that DOX not only decreased tumor cell numbers but also reduced F-SPION uptake. Testing equal numbers of treated cells revealed further signal decline with longer DOX exposure, underscoring the importance of uptake capacity and physiological state. *In vivo*, the tumor signal did not significantly change in the DOX-treated mice between Day 1 and Day 7, whereas the control mice exhibited an approximately 100% increase. The signal gap increased from 54 pT to 405 pT, demonstrating that SERF plus F-SPION can detect early tumor responses to chemotherapy.

In comparison with conventional imaging techniques, our method demonstrates several unique strengths. MRI and CT provide mainly structural information and often fail to capture early functional changes in tumors. Nuclear medicine imaging can detect metabolic alterations, but its reliance on ionizing radiation makes it less suitable for repeated or long-term use. SQUID-based magnetometry achieves high sensitivity but requires cryogenic cooling, which limits its practicality 46. In contrast, the use of SERF magnetometry combined with F-SPION allows real-time, radiation-free functional monitoring at room temperature, making it well suited for longitudinal evaluation. This approach is not without limitations. SERF magnetometers are extremely sensitive to background magnetic noise, meaning that shielding or advanced signal processing is necessary in clinical environments 47. In addition, the spatial resolution of this technique is lower than those of MRI and CT, suggesting that this approach will be most effective when used in combination with anatomical imaging. Another consideration is that F-SPION uptake may vary depending on the tumor microenvironment, including vascularization and phagocytic activity, which could affect measurement sensitivity across tumor types. Despite these challenges, the method holds strong translational promise. In addition to detecting early therapeutic responses, as shown in this study, it could also be applied to long-term tumor monitoring without the risk of cumulative radiation, and in pediatric oncology, where minimizing radiation exposure is especially important.

## Conclusion

This study developed and validated F-SPION, a novel nanoparticle with combined ferromagnetic and superparamagnetic properties, with long-term intracellular magnetic retention, capable of enhancing tumor signals while maintaining stability and generating minimal background noise. F-SPION-mediated signal enhancement depends on cellular uptake and magnetic activation, providing strong and persistent signal contrast between tumor and normal tissues for at least 1 hour. The changes in the signal intensity of the tumor reflect the response of the tumor to treatment, including a reduction in tumor cell count and a decrease in uptake ability. Combined with highly sensitive SERF detection technology, F-SPION allows noninvasive and real-time monitoring of therapeutic efficacy at the cellular level, offering an alternative to current tumor morphology-based methods and expanding the scope of human magnetic field research.

## Supplementary Material

Supplementary figures.

## Figures and Tables

**Scheme 1:**
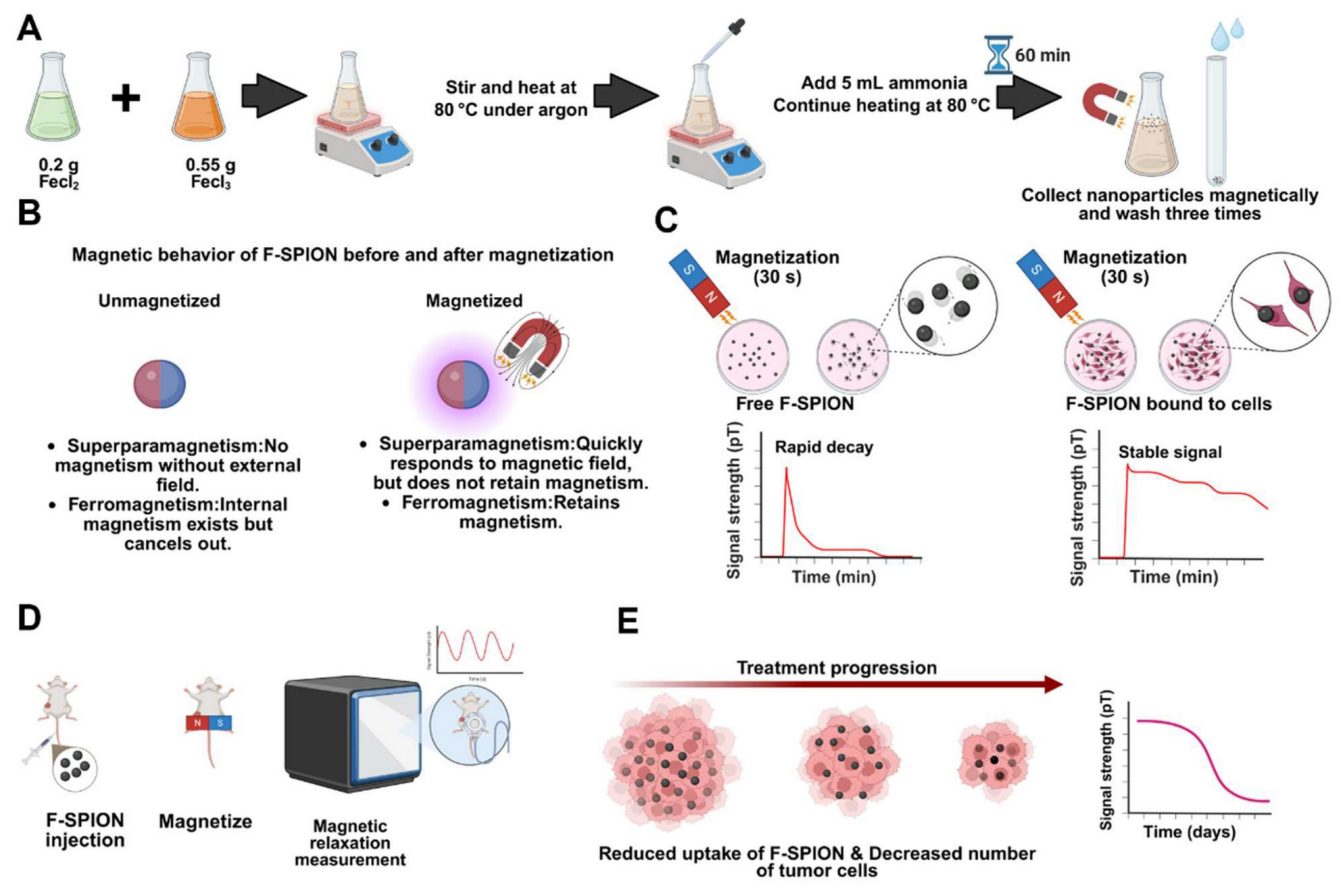
** Schematic illustration of the synthesis, magnetic behavior, and application of F-SPION for tumor signal detection and therapeutic monitoring.** A) Synthesis of F-SPION. B) Magnetic behavior of F-SPION before and after magnetization. C) Magnetic signal behavior comparison between free F-SPION and cell-bound F-SPION. D) *In vivo* workflow for F-SPION injection, magnetization, and magnetic relaxation measurements under zero magnetic field conditions. E) Decrease in magnetic signal intensity during tumor treatment progression.

## Abbreviations

CT: computed tomography
DAPI: 4′,6-diamidino-2-phenylindole
DLS: dynamic light scattering
DOX: doxorubicin
FOV: field of view
F-SPION: ferromagnetic-superparamagnetic iron oxide nanoparticle
FTIR: Fourier transform infrared spectroscopy
FITC: fluorescein isothiocyanate
FFT: fast Fourier transform
H&E: hematoxylin and eosin
Hc: coercivity
ICP-MS: inductively coupled plasma mass spectrometry
LPS: lipopolysaccharide
MRI: magnetic resonance imaging
Mr: remanence
Ms: saturation magnetization
PBS: phosphate-buffered saline
RBC: red blood cell
SERF: spin-exchange relaxation-free
T_2_WI: T_2_-weighted imaging
TR: repetition time
VSM: vibrating sample magnetometer
XPS: X-ray photoelectron spectroscopy
XRD: X-ray diffraction
