# Supplementary material for: High-sensitivity spin-exchange relaxation-free (SERF) magnetometry combined with magnetic-responsive iron oxide nanoparticles for real-time monitoring of malignant tumor therapy
Source: Theranostics. 2026 Jan 1;16(3):1123–42. doi: 10.7150/thno.117893 (PMC12679083; doi:10.7150/thno.117893)
Supplement: Supplementary file 1 — Supplementary figures. [file thnov16p1123s1.pdf]

Supplementary Information*

High-sensitivity spin-exchange relaxation-free (SERF) magnetometry combined with magnetic-responsive iron oxide nanoparticles for real-time monitoring of malignant tumor therapy

Wenbo Wang[1,7]#, Gaorui Zhang[2]#, Baosheng Wang[3], Dawei Zhou[1], Ning Ding[4], Ziyuan Huang[3,4], Shiqiang Zheng[3,4], Qichao Cheng[1], Mingchuan Yu[6], Min Xiang[3,4]*, Yuguo Chen[7,8]*, Jiazhi Duan[5]*, Dexin Yu[1,7]*

[1] Department of Radiology, Qilu Hospital of Shandong University, Jinan, Shandong, 250012, China.
[2] Department of Radiology, Weifang People's Hospital, Shandong Second Medical University, Weifang, China.
[3] National Institute of Extremely-Weak Magnetic Field Infrastructure, Hangzhou 310028, China.
[4] School of Instrumentation and Optoelectronic Engineering, Beihang University, Beijing 100083, China.
[5] Institute for Advanced Interdisciplinary Research, University of Jinan, Jinan 250022, China.
[6] Department of Rehabilitation Medicine, the Second Affiliated Hospital of Nanchang University, Nanchang 330006, P. R. China.
[7] Interdisciplinary Center, Shandong University, Jinan 250100, China.
[8] Department of Emergency Medicine, Qilu Hospital of Shandong University, Jinan 250012, China.

* Corresponding authors.

[#]These authors contributed equally to this work.

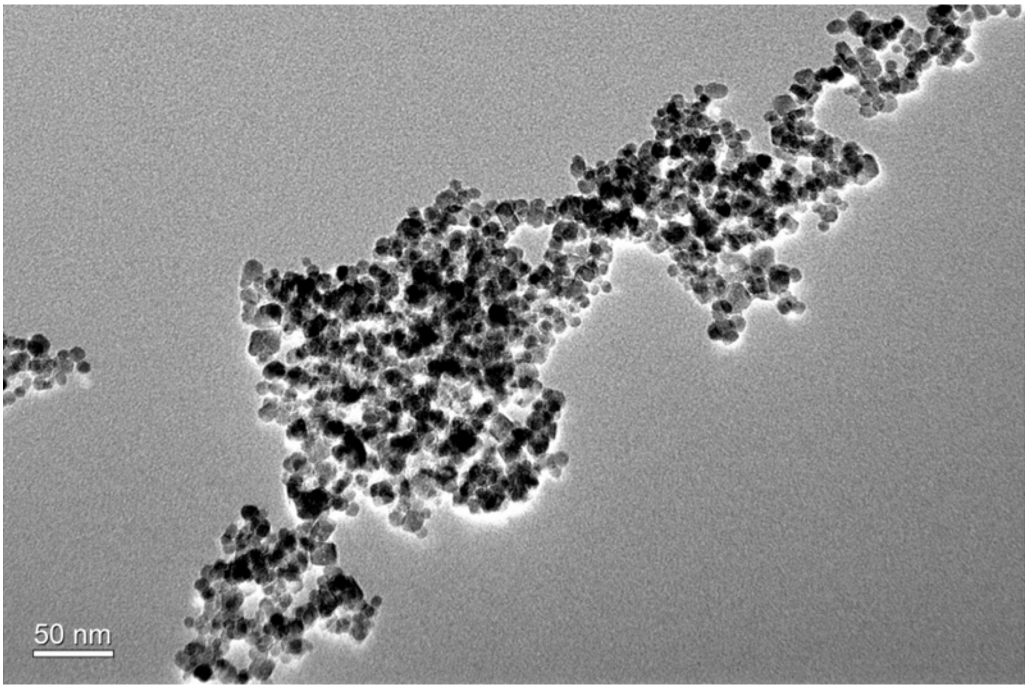

Figure S1. High-resolution transmission electron microscopy image of F-SPION.

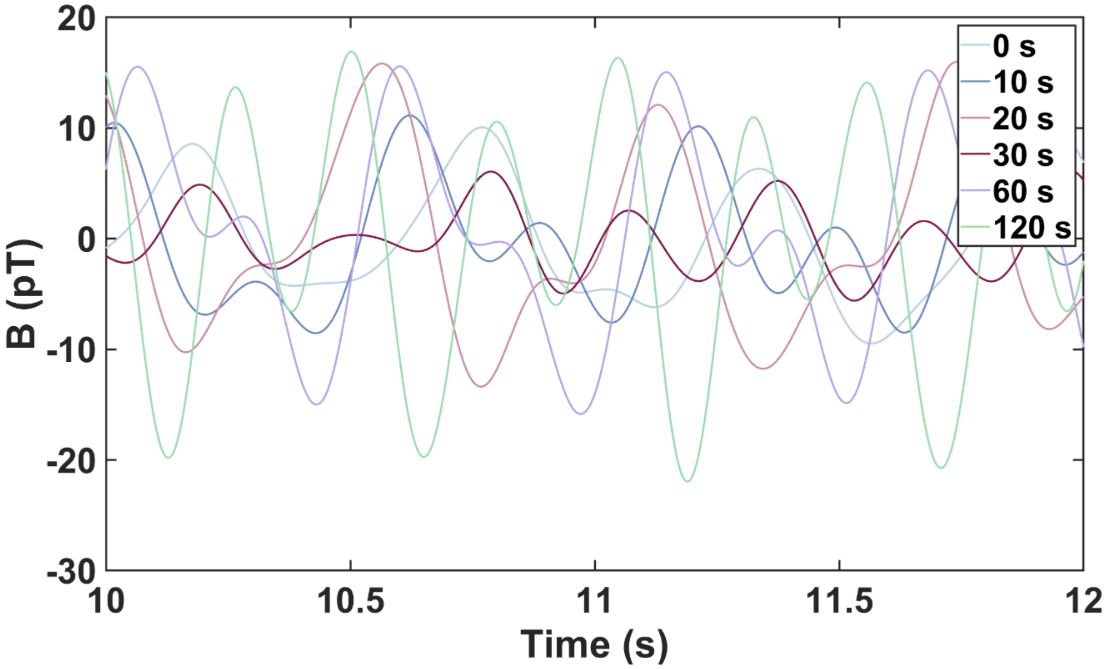

Figure S2. Magnetic signals of F-SPION at a fixed concentration of 100 μg/mL after different durations of magnetization (0, 10, 20, 30, 60, and 120 s). Signal intensity increased with prolonged magnetization time, reflecting enhanced magnetic responsiveness of F-SPION.

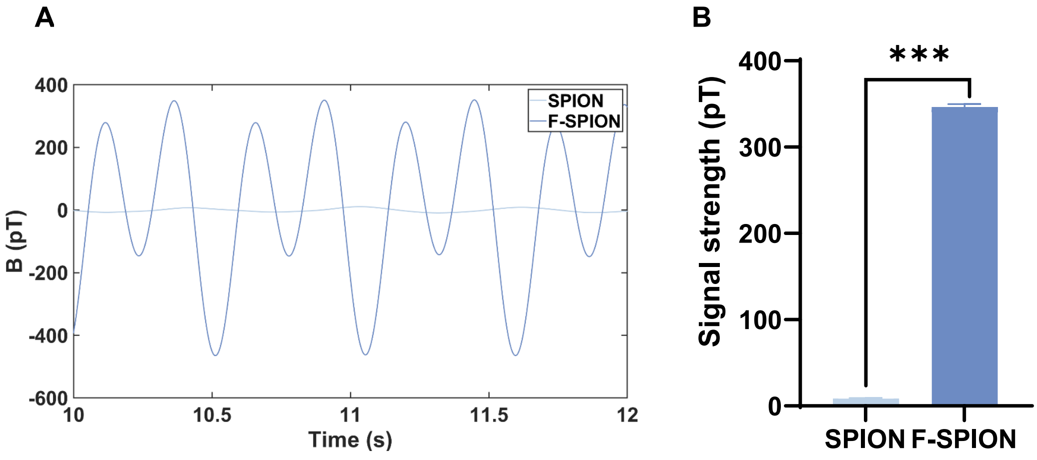

Figure S3. Magnetic signal of 4T1 cells incubated with SPION or F-SPION for 6 h followed by magnetization. A) Magnetic signals of SPION- and F-SPION-treated 4T1 cells. B) Statistical comparison of signal intensities of SPION- and F-SPION-treated 4T1 cells. The data are presented as the mean ± SD (n = 5). Statistical analysis was performed using an unpaired two-tailed Student's *t*-test. ***$P < 0.001$.

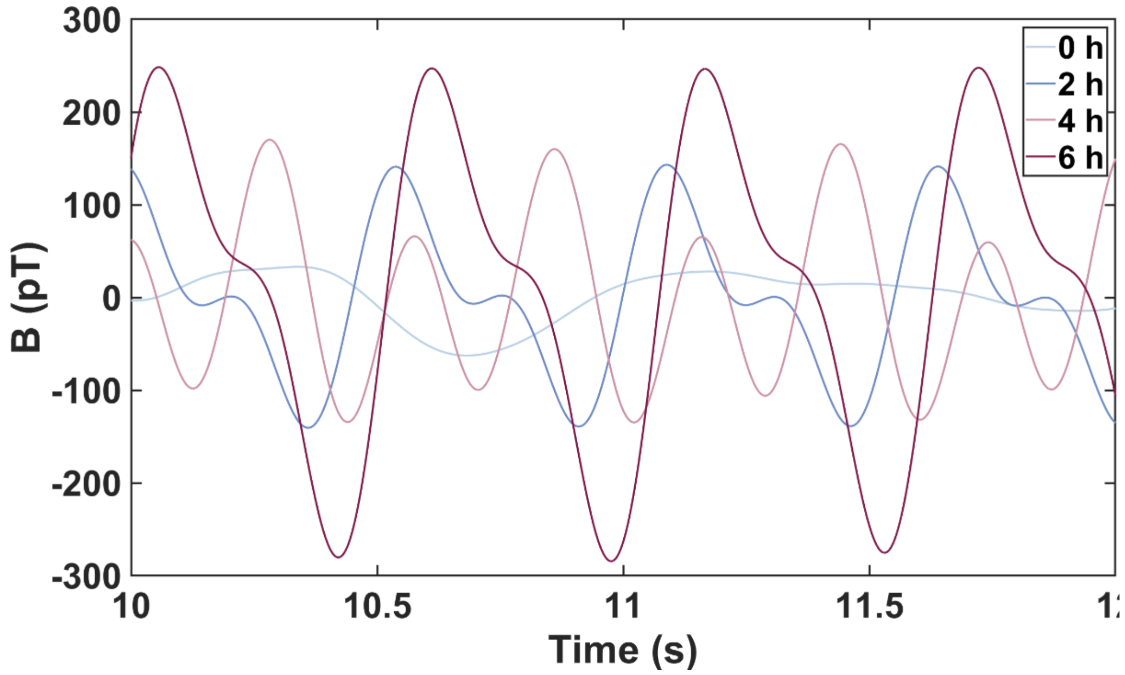

Figure S4. Magnetic signals of F-SPION after co-incubation with 4T1 cells for different durations (0, 2, 4, and 6 h), followed by 30 s of magnetization and measurement 30 min after magnetization. Longer incubation times led to stronger magnetic signals.

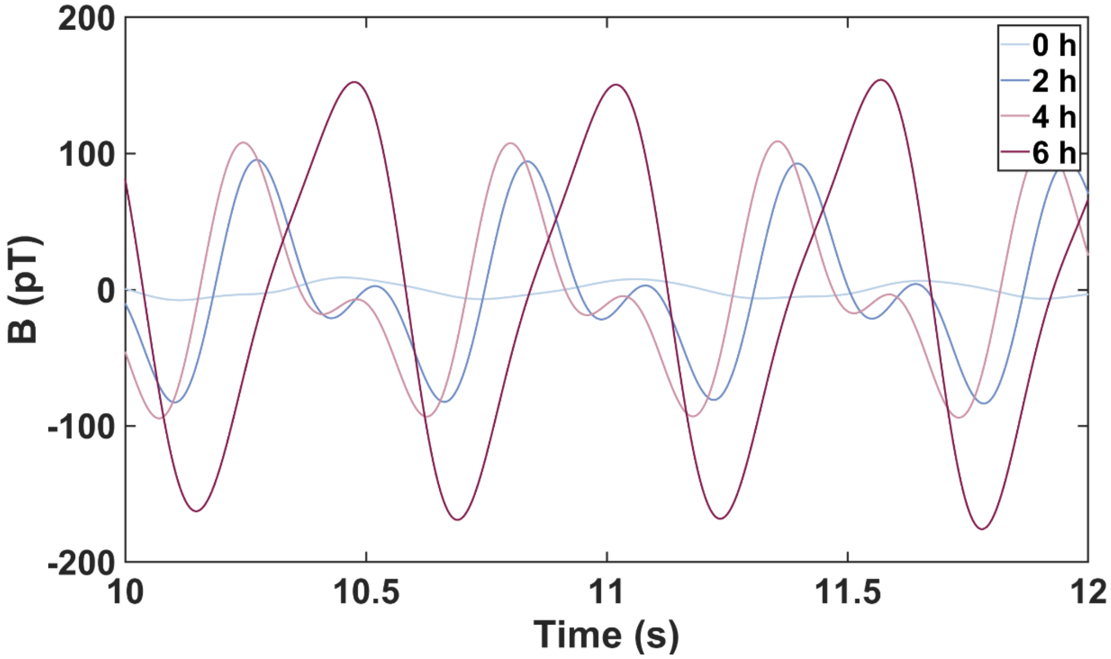

Figure S5. Magnetic signals of F-SPION after co-incubation with 4T1 cells for different durations (0, 2, 4, and 6 h), followed by 30 s of magnetization and measurement 60 min after magnetization. Longer incubation times led to stronger magnetic signals.

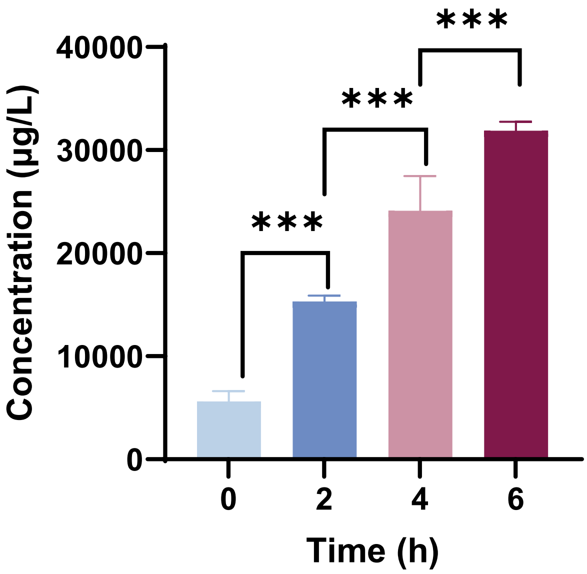

Figure S6. ICP-MS analysis of intracellular iron concentration in 4T1 cells at different incubation times with F-SPION. The data are presented as the mean ± standard deviation (n = 5). Statistical significance was analyzed using one-way ANOVA, ****P* < 0.001.

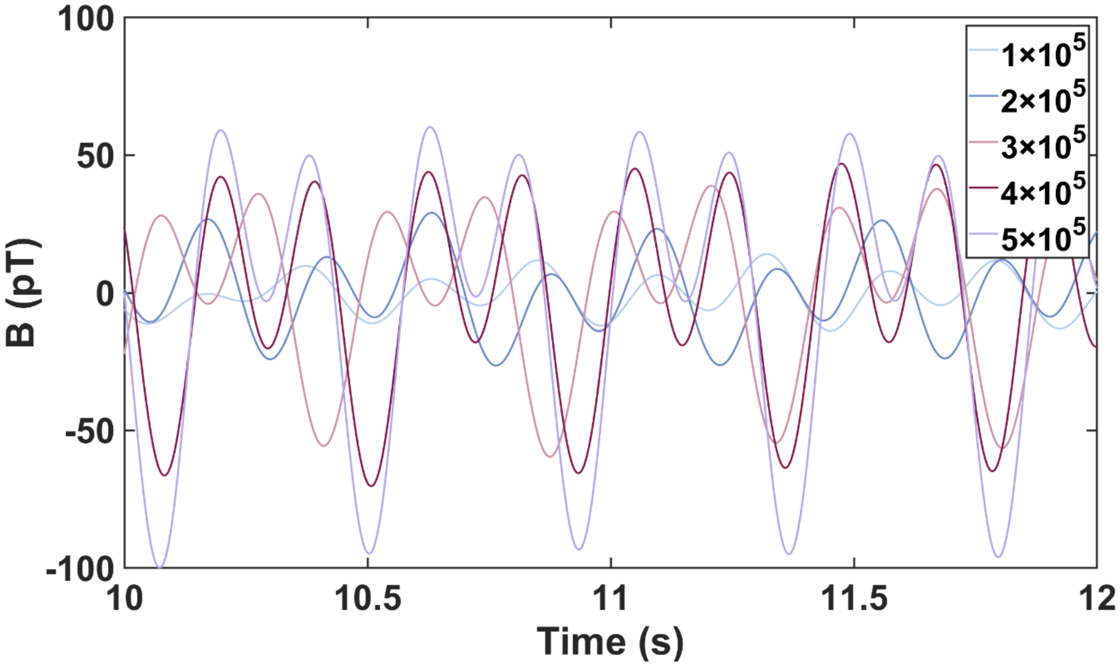

Figure S7. Magnetic signals of different numbers of 4T1 tumor cells after incubation with F-SPION and subsequent 30 s of magnetization. Signal intensity increased with higher cell numbers, reflecting the positive correlation between magnetic signal strength and tumor cell quantity.

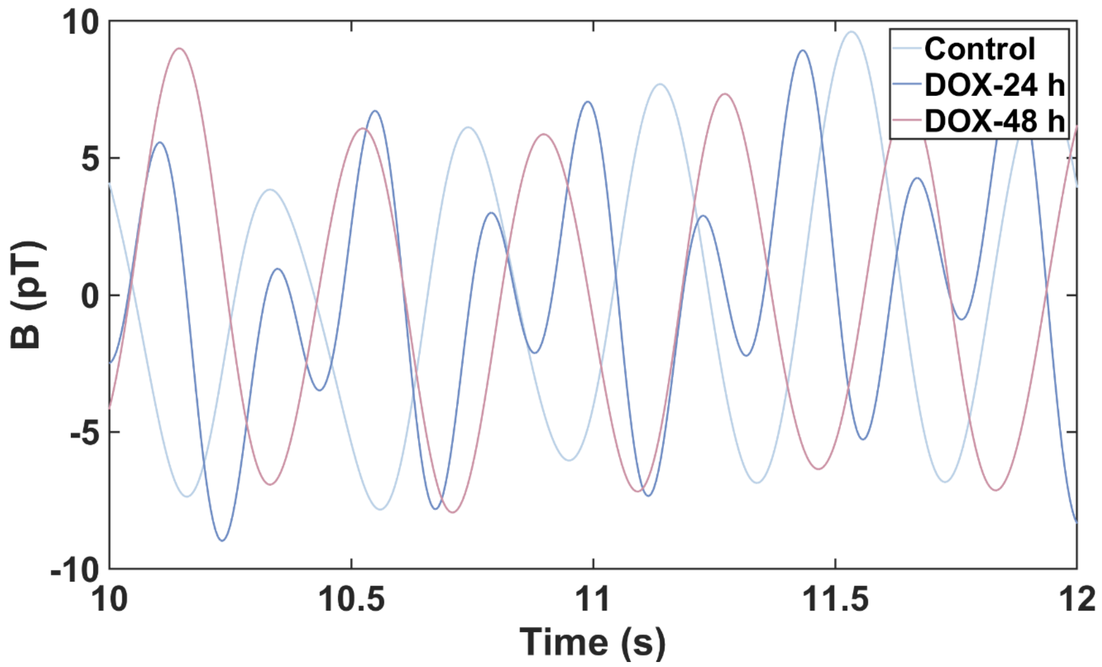

Figure S8. Magnetic signals of 4T1 cells in control and DOX-treated groups (24 h and 48 h) before magnetization. No adjustment for cell number was performed after DOX treatment.

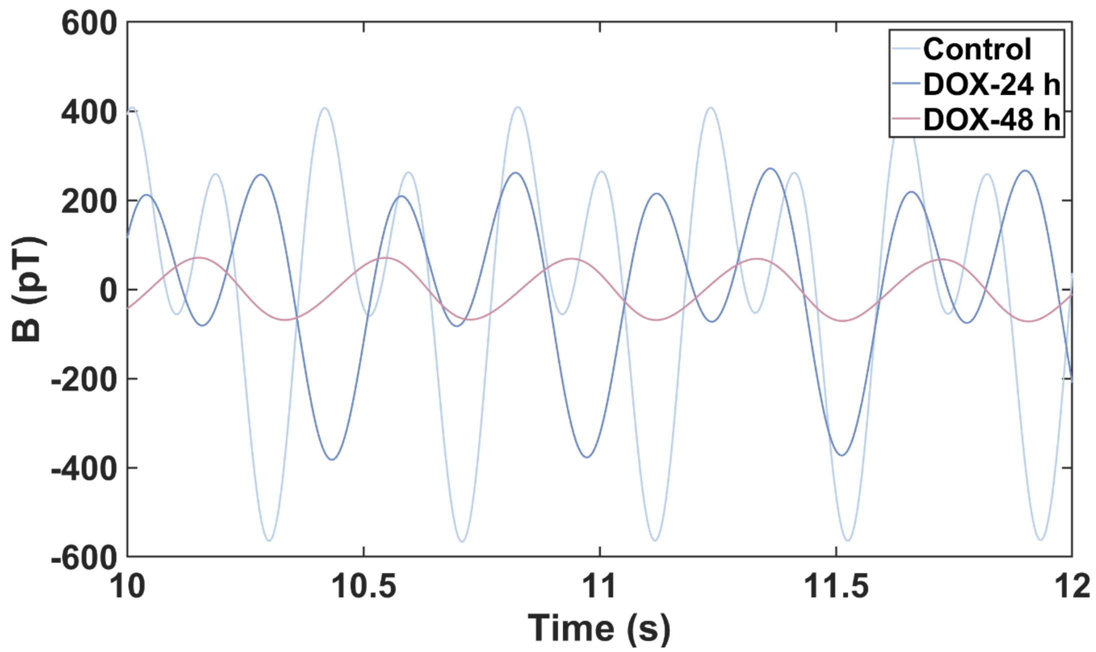

Figure S9. Magnetic signals of 4T1 cells in control and DOX-treated groups (24 h and 48 h) after 30 s of magnetization. No adjustment for cell number was performed after DOX treatment. The decrease in magnetic signal intensity reflects both DOX-induced tumor cell damage and reduced cell numbers.

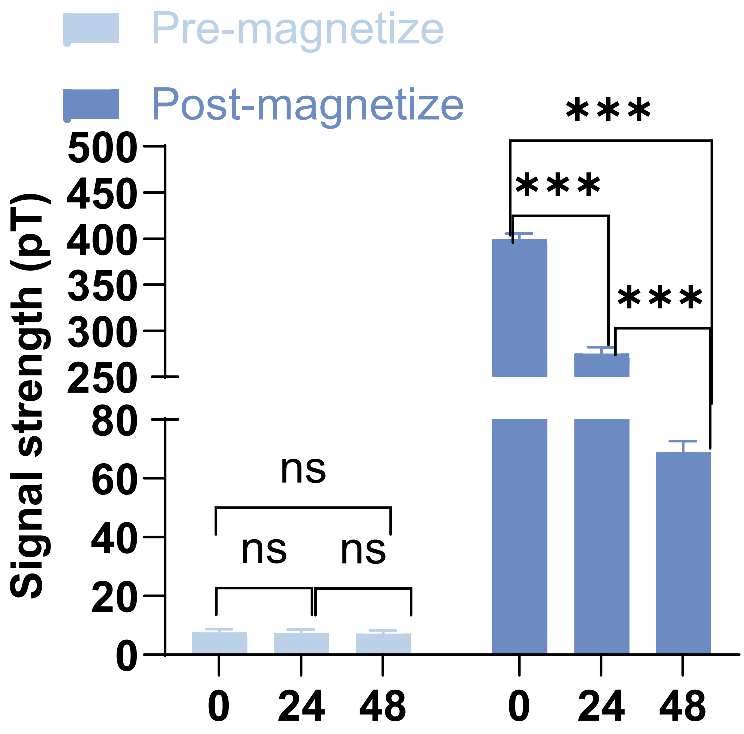

Figure S10. Statistical analysis of magnetic signal intensities in 4T1 cells treated with DOX for 0, 24, and 48 h before and after magnetization. No adjustment for cell number was performed after DOX treatment. The data are presented as the mean ± standard deviation (n = 5). Statistical significance was analyzed by one-way ANOVA; ns, not significant; ****P* < 0.001.

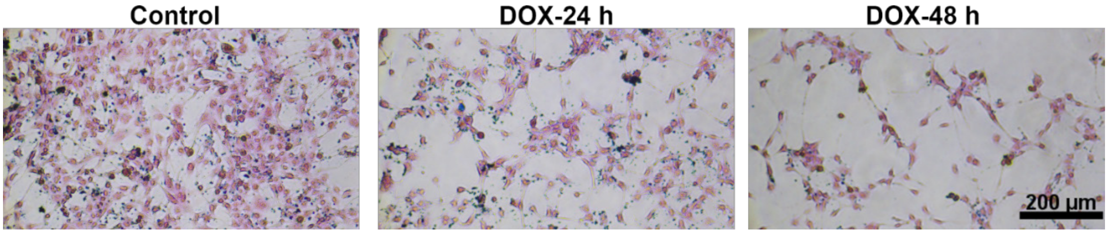

Figure S11. Prussian blue staining of 4T1 cells after co-incubation with F-SPION and treatment with DOX for 0, 24, and 48 h. The extent of intracellular iron accumulation decreased with prolonged DOX exposure, reflecting reduced cellular uptake capacity. Scale bar = 200 μm.

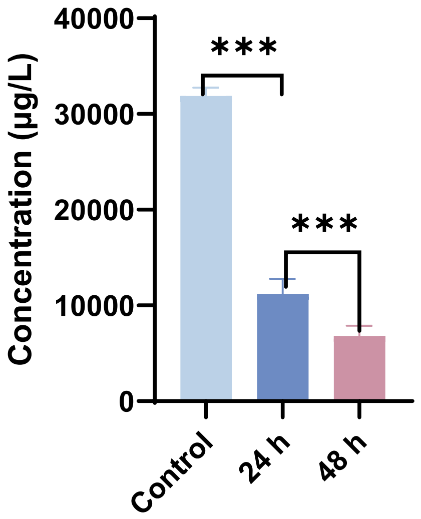

Figure S12. ICP-MS analysis of intracellular iron concentration in 4T1 cells after co-incubation with F-SPION and DOX at different time points. The data are presented as the mean ± standard deviation (n = 5). Statistical significance was analyzed by one-way ANOVA; ns, not significant; ****P* < 0.001.

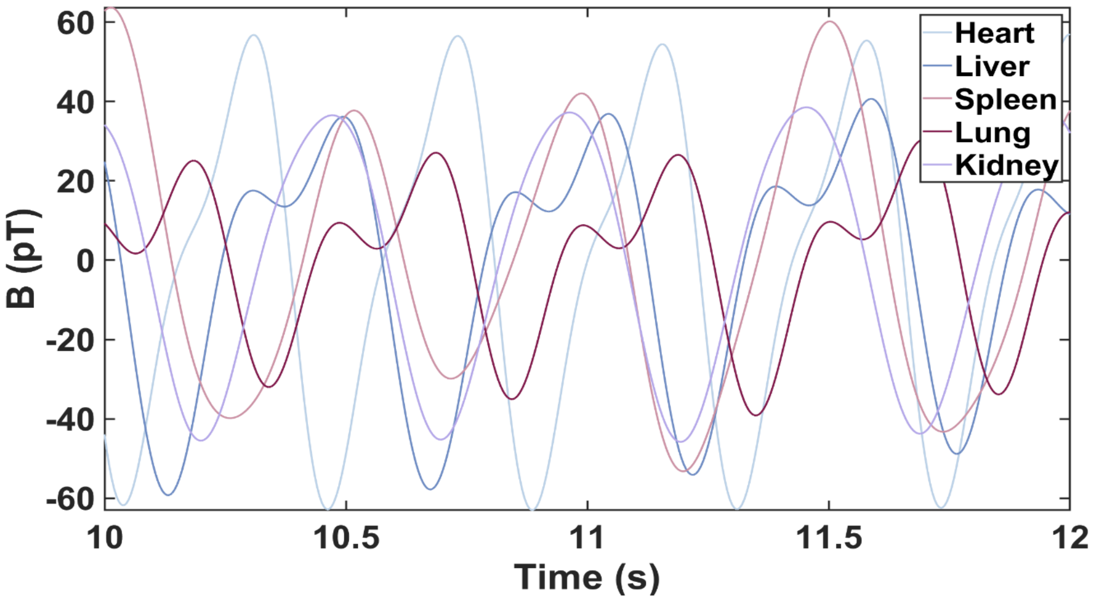

Figure S13. Magnetic signals of major organs (heart, liver, spleen, lung, and kidney) from mice without F-SPION injection after 30 s of magnetization. These results reflect the baseline magnetic background of normal tissues without nanoparticle labeling.

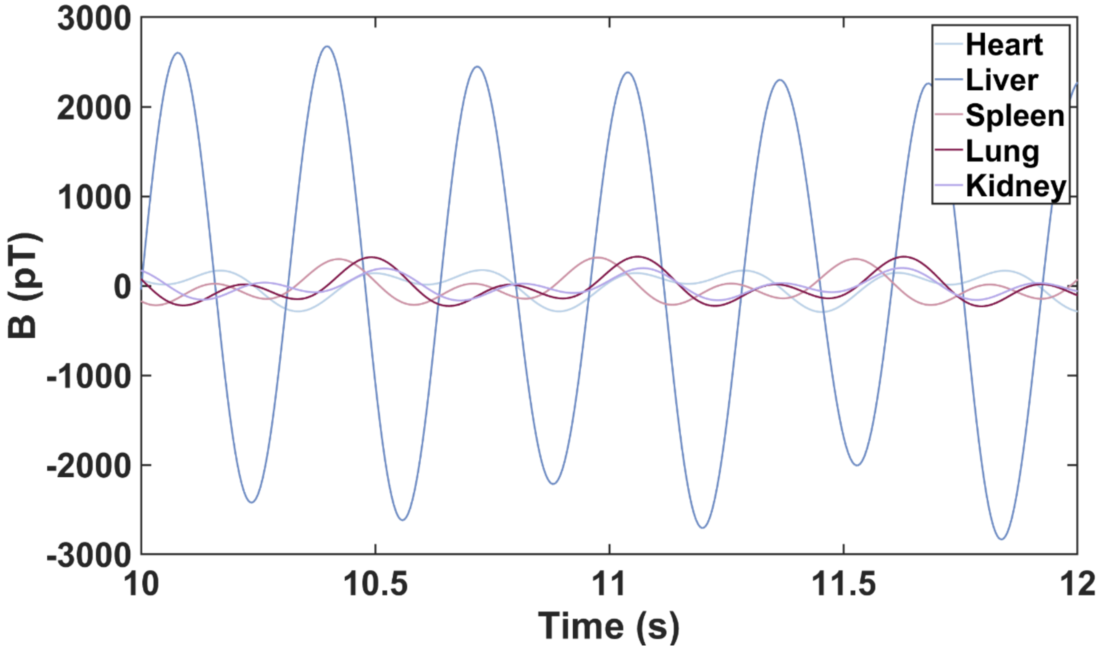

Figure S14. Magnetic signals of major organs (heart, liver, spleen, lung, and kidney) from mice 30 min after F-SPION injection and 30 s of magnetization. The liver exhibited the strongest magnetic signal.

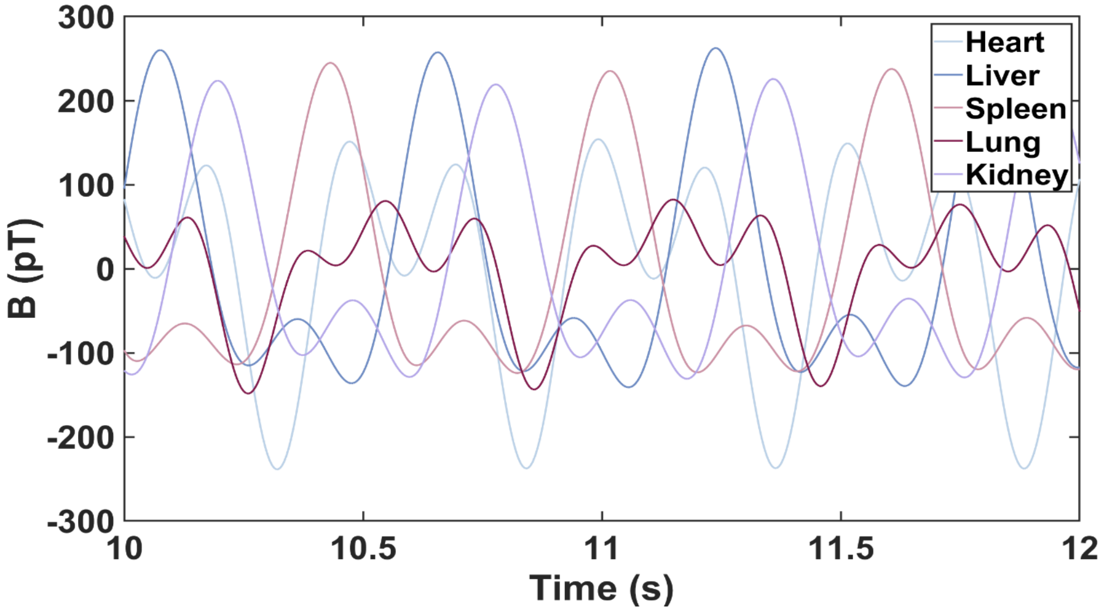

Figure S15. Magnetic signals of major organs (heart, liver, spleen, lung, and kidney) from mice 60 min after F-SPION injection and 30 s of magnetization. The liver exhibited the strongest magnetic signal, followed by the spleen and lung.

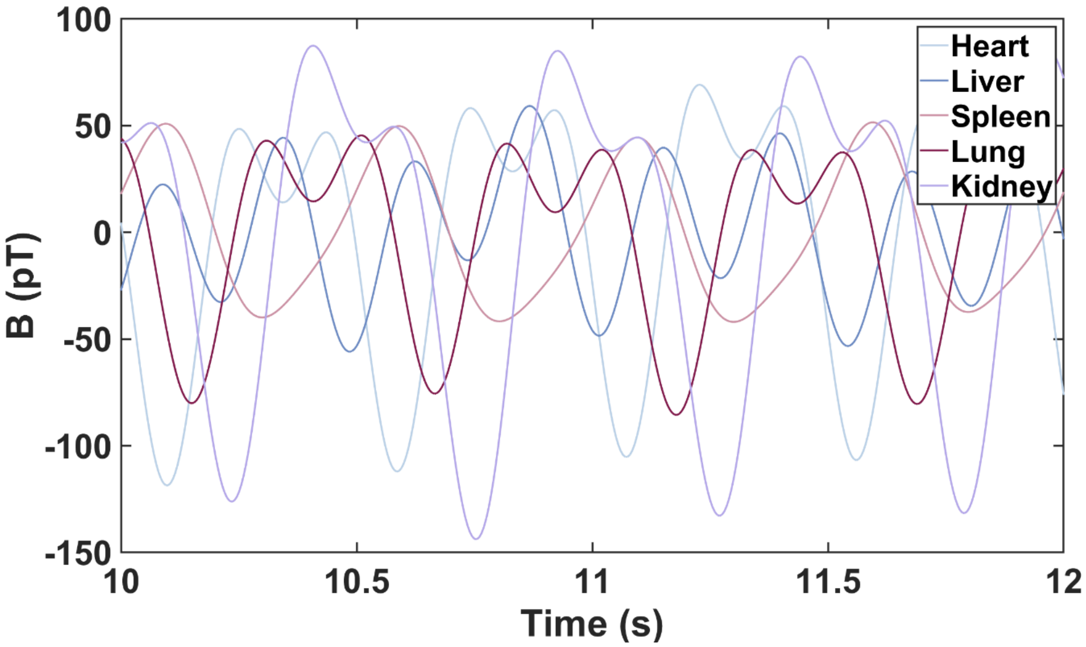

Figure S16. Magnetic signals of major organs (heart, liver, spleen, lung, and kidney) from mice 90 min after F-SPION injection and 30 s of magnetization. The kidney exhibited the strongest magnetic signal.

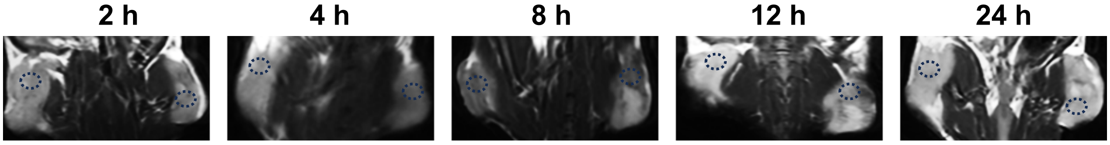

Figure S17. MR images of tumor-bearing mice at different times post-magnetization. The dotted circles indicate the regions of interest used for quantifying tumor enhancement values.

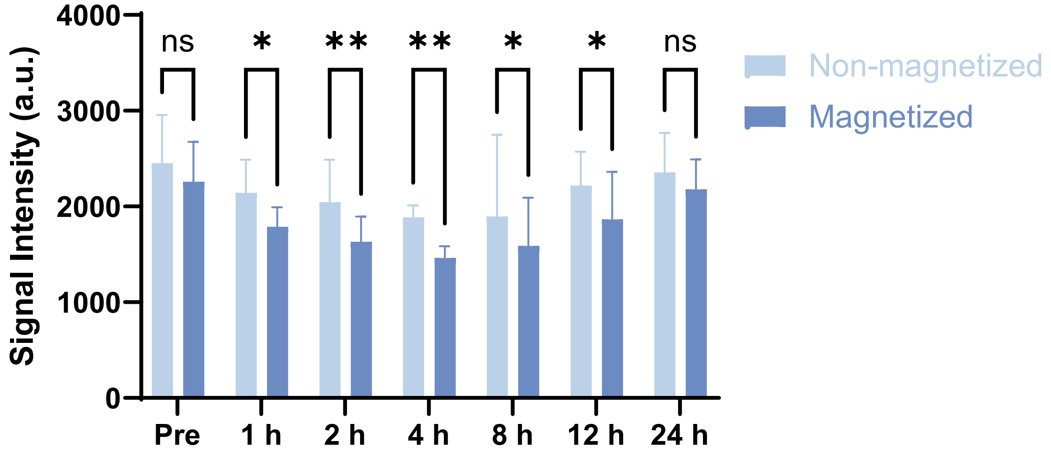

Figure S18. Signal intensity measurements in tumor tissues at different time points under magnetized and non-magnetized conditions. The data are presented as the mean ± standard deviation (n = 5). Statistical significances were analyzed by paired t-test; ns, not significant; *$P < 0.05$; **$P < 0.01$.

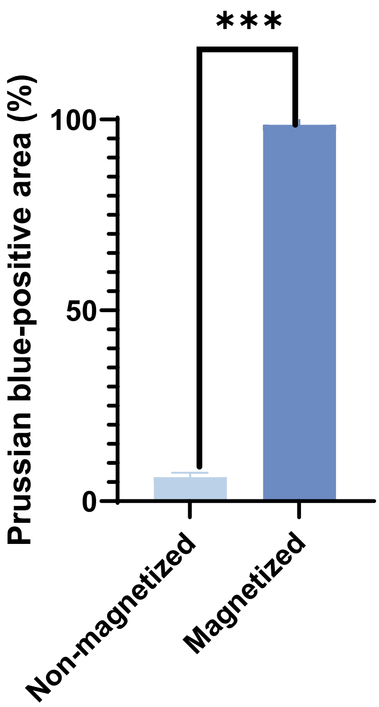

Figure S19. Quantification of Prussian blue-positive area in tumor sections from non-magnetized and magnetized tumors. The percentage of Prussian blue-positive area was calculated relative to the total tissue area in paraffin-embedded sections. The Data are presented as the mean ± SD (n = 5). Statistical analysis was performed using a paired two-tailed Student's *t*-test. ***$P < 0.001$.

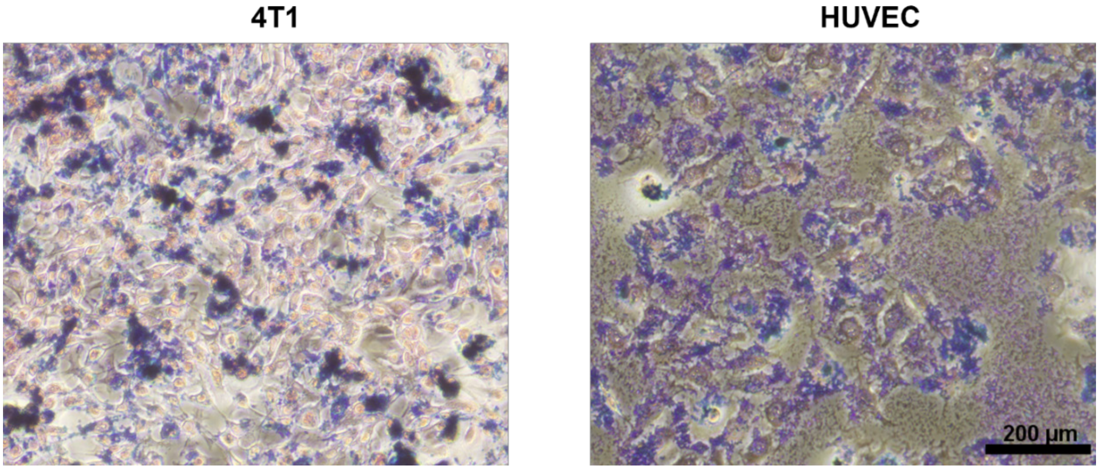

Figure S20. Prussian blue staining of 4T1 tumor cells (left) and HUVECs (right) after co-incubation with F-SPION (100 µg/mL, 6 h).

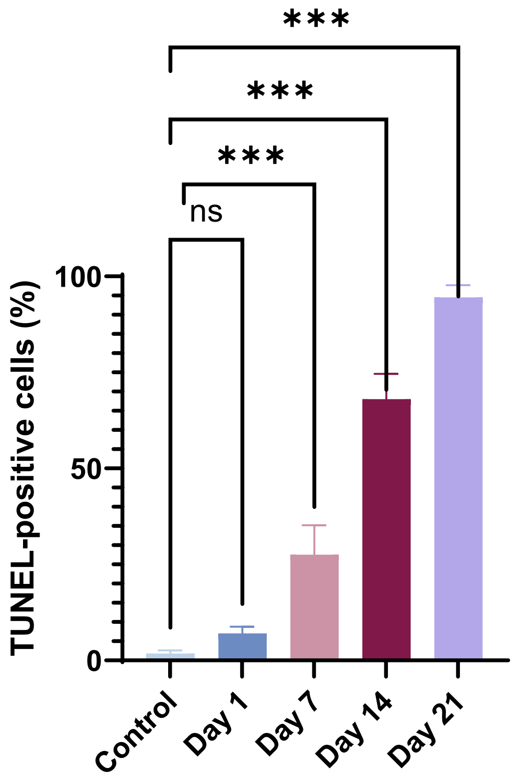

Figure S21. Quantification of TUNEL-positive cells in tumor tissues among different groups. Representative sections were analyzed, and the percentage of TUNEL-positive cells was calculated relative to the total number of DAPI-stained nuclei. The data are presented as the mean ± SD (n = 5). Statistical analysis was performed using one-way ANOVA followed by Tukey’s post hoc test. ns, not significant; ****P* < 0.001.

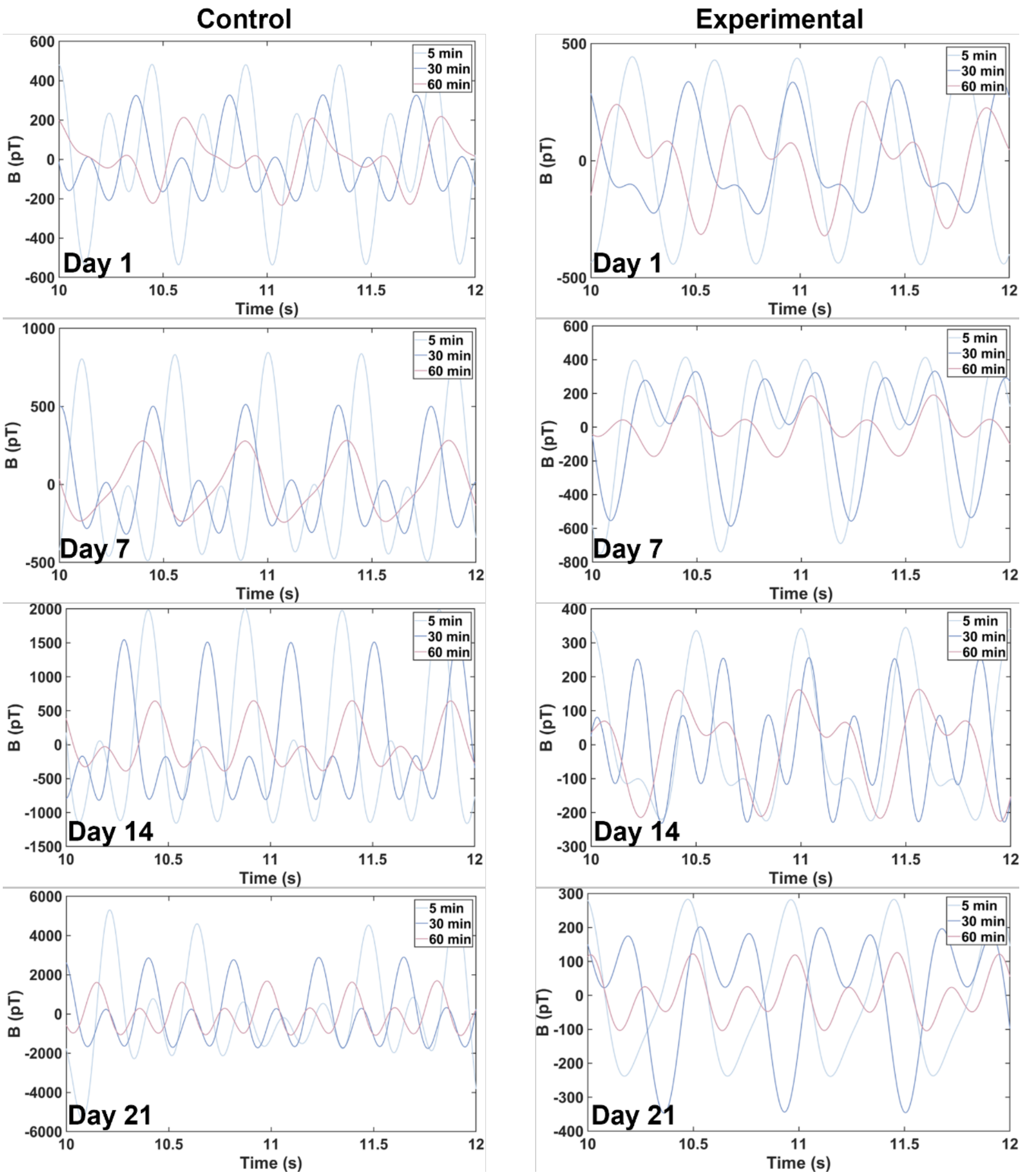

Figure S22. Signal intensities of tumor tissues in control and experimental groups at different time points.
